# Supplementary material for: Public attitudes in England towards the sharing of personal data following a mass casualty incident: a cross-sectional study
Source: BMJ Open. 2018 May 20;8(5):e022852. doi: 10.1136/bmjopen-2018-022852 (PMC5961558; doi:10.1136/bmjopen-2018-022852)
Supplement: Supplementary file 1 [file bmjopen-2018-022852supp001.pdf]

KCL Disaster Data Protection Survey – Wave 2
Topline results

- Results are based on interviews with 1000 members of the public aged 18-65 across England. Interviews were conducted between 3 and 10 July 2017.
- All interviews were conducted via Ipsos MORI's online panel.
- The survey data is unweighted but based on quotas on age, gender, region and working status (with age and gender interlocked) to reflect the profile of adults aged 18-65 in England, with up to 10% flexibility on region and working status. Weights are available in the SPSS file for the whole sample, Sample A and Sample B, for both the first and second wave and for both waves combined.
- Results are based on all respondents (1000) unless otherwise stated. Bases specified are unweighted.
- Where percentages do not sum to 100, this may be due to respondents being able to select multiple responses, computer rounding or the exclusion of 'don't know'/ not stated.
- An asterisk (*) represents a value of less than half of one percent, but greater than zero.
- 14,735 people were invited to take part in the survey.
- 30 participants ended the survey without completing it (and were not included in the data); 19 people were excluded from the data due to being identified as 'speeding' or straightlining'; i.e. completing the survey too quickly to have given genuine, considered answers, or providing identical answers to five or more consecutive questions where this was possible.
- This work has been carried out in accordance with the requirements of the international quality standard for Market Research, ISO 20252:2012, and with the standard Ipsos MORI Terms and Conditions which can be found at http://www.ipsos-mori.com/terms. © Ipsos MORI 2017

Q1. Please select your date of birth (recoded into age categories):

	%
18-24	14.4
25-34	22.2
35-44	20.7
45-54	22.6
55-65	20.1

Q2. Please state the gender you identify yourself with:

	%
Male	49.6
Female	50.4

Q3. In which of the following regions do you live?

	%
North East	4.6
North West	13.5
Yorkshire and Humberside	10.1
West Midlands	10.1
East Midlands	8.4
East of England	9.9
South West	9.0
South East	16.6

London	17.8

Q4. Which of the following best describes your employment status?

	%
Working – full time (30 or more hours a week)	50.9
Working – part-time (less than 30 hours a week)	14.0
Self-employed	7.8
Unemployed – looking for a job	5.2
Unemployed – not looking for a job/long-term sick or disabled/Housewife/husband/Full-time carer	10.3
Retired	5.6
Pupil/Student/In full-time education	6.2
**NET: Working**	72.7
**NET: Not working**	27.3

We are interested in people's views about sharing information following a major incident such as a terrorist attack. The researchers have no reason to think that an attack is going to happen. This research is one of a number of studies that academics at King's College London are conducting on how people might react to a range of different issues.

SAMPLE A:
Imagine that you are on holiday in another country and witness a terrorist shooting. Some people are badly injured but you are not harmed and neither is anyone you know well. The British government arranges for you to be flown home, along with other British nationals who were in the area. When you arrive back in Britain, a police officer at the airport records your name, address, phone number and email address. The following questions ask about things that might happen next.

SAMPLE B:
Imagine that you are on holiday in another country and a place you visit is discovered by police to be contaminated by radioactive material. The police believe this is linked to a terrorist group. Some people are badly injured but you are not harmed and neither is anyone you know well. The British government arranges for you to be flown home, along with other British nationals who were in the area. When you arrive back in Britain, a police officer at the airport records your name, address, phone number and email address. The following questions ask about things that might happen next.

Q5. Following an incident like this, various groups or individuals might ask the police to tell them who was caught up in the incident. Please say whether you think that the police are or are not currently able to share your name and contact details with these groups or people following an incident like this, without checking with you first.

1. **Your GP, so they can check how you are doing**

	% (TOTAL – Base = 1000)	% (SAMPLE A – Base = 500)	% (SAMPLE B – Base = 500)
Definitely able to share	19.6	19.8	19.4
Probably able to share	34.1	28.8	39.4
Not sure	20.9	21.2	20.6
Probably not able to share	15.2	18.8	11.6
Definitely not able to share	10.2	11.4	9.0

2. **Your travel insurance company, so they can offer you practical and financial support**

	% (TOTAL – Base = 1000)	% (SAMPLE A – Base = 500)	% (SAMPLE B – Base = 500)
Definitely able to share	10.6	11.8	9.4
Probably able to share	22.9	22.4	23.4
Not sure	24.6	24.2	25.0
Probably not able to share	23.2	22.8	23.6

Definitely not able to share	18.7	18.8	18.6

3. A team of medical researchers from a university, so they can invite you to take part in a study to improve the way future incidents are dealt with

	% (TOTAL – Base = 1000)	% (SAMPLE A – Base = 500)	% (SAMPLE B – Base = 500)
Definitely able to share	4.4	3.8	5.0
Probably able to share	13.2	10.6	15.8
Not sure	23.7	23.6	23.8
Probably not able to share	30.1	31.4	28.8
Definitely not able to share	28.6	30.6	26.6

4. A journalist, so they can write a news article about the incident

	% (TOTAL – Base = 1000)	% (SAMPLE A – Base = 500)	% (SAMPLE B – Base = 500)
Definitely able to share	2.3	2.4	2.2
Probably able to share	6.3	6.2	6.4
Not sure	14.1	13.2	15.0
Probably not able to share	20.7	19.0	22.4
Definitely not able to share	56.6	59.2	54.0

5. A charity, such as the British Red Cross, so they can offer you support

	% (TOTAL – Base = 1000)	% (SAMPLE A – Base = 500)	% (SAMPLE B – Base = 500)
Definitely able to share	5.7	5.6	5.8
Probably able to share	21.1	20.0	22.2
Not sure	27.2	27.6	26.8
Probably not able to share	26.5	25.6	27.4
Definitely not able to share	19.5	21.2	17.8

6. A health-related Government organisation, so they can send you a questionnaire to find out if you might benefit from extra care or support.

	% (TOTAL – Base = 1000)	% (SAMPLE A – Base = 500)	% (SAMPLE B – Base = 500)
Definitely able to share	10.7	11.0	10.4
Probably able to share	34.9	29.8	40.0
Not sure	26.6	28.0	25.2
Probably not able to share	17.1	19.4	14.8
Definitely not able to share	10.7	11.8	9.6

7. A specialist NHS team, to provide you with information about ways to get support for any physical or mental health issues.

	% (TOTAL – Base = 1000)	% (SAMPLE A – Base = 500)	% (SAMPLE B – Base = 500)
Definitely able to share	19.4	19.8	19.0
Probably able to share	41.4	38.0	44.8
Not sure	20.4	19.0	21.8
Probably not able to share	12.4	15.6	9.2
Definitely not able to share	6.4	7.6	5.2

8. A law firm, so they can offer to represent you in a 'no-win, no-fee' claim for compensation

	% (TOTAL – Base = 1000)	% (SAMPLE A – Base = 500)	% (SAMPLE B – Base = 500)
Definitely able to share	3.8	5.0	2.6
Probably able to share	9.0	8.4	9.6
Not sure	17.9	16.0	19.8
Probably not able to share	25.1	26.2	24.0
Definitely not able to share	44.2	44.4	44.0

Q6. Following an incident like this, various groups or individuals might ask the police to tell them who was caught up in the incident. For each of the following groups, please say whether you think that is acceptable or not acceptable for the police to share your name and contact details with these groups or people following an incident like this, without checking with you first;

1. Your GP, so they can check how you are doing

	% (TOTAL – Base = 1000)	% (SAMPLE A – Base = 500)	% (SAMPLE B – Base = 500)
Definitely acceptable to share	30.1	28.6	31.6
Probably acceptable to share	41.0	41.6	40.4
Not sure	13.0	12.0	14.0
Probably not acceptable to share	8.0	8.4	7.6
Definitely not acceptable to share	7.9	9.4	6.4

2. Your travel insurance company, so they can offer you practical and financial support

	% (TOTAL – Base = 1000)	% (SAMPLE A – Base = 500)	% (SAMPLE B – Base = 500)
Definitely acceptable to share	10.8	12.2	9.4
Probably acceptable to share	28.8	28.0	29.6
Not sure	20.4	20.0	20.8
Probably not acceptable to share	17.7	19.4	16.0
Definitely not acceptable to share	22.3	20.4	24.2

3. A team of medical researchers from a university, so they can invite you to take part in a study to improve the way future incidents are dealt with

	% (TOTAL – Base = 1000)	% (SAMPLE A – Base = 500)	% (SAMPLE B – Base = 500)
Definitely acceptable to share	4.9	3.4	6.4
Probably acceptable to share	17.7	16.2	19.2
Not sure	24.5	22.4	26.6
Probably not acceptable to share	26.7	30.2	23.2
Definitely not acceptable to share	26.2	27.8	24.6

4. A journalist, so they can write a news article about the incident

	% (TOTAL – Base = 1000)	% (SAMPLE A – Base = 500)	% (SAMPLE B – Base = 500)
Definitely acceptable to share	1.8	2.0	1.6
Probably acceptable to share	4.8	4.2	5.4
Not sure	10.1	7.4	12.8
Probably not acceptable to share	15.6	15.4	15.8
Definitely not acceptable to share	67.7	71.0	64.4

5. A charity, such as the British Red Cross, so they can offer you support

	% (TOTAL – Base = 1000)	% (SAMPLE A – Base = 500)	% (SAMPLE B – Base = 500)
Definitely acceptable to share	7.2	7.0	7.4
Probably acceptable to share	29.8	28.8	30.8
Not sure	25.1	25.4	24.8
Probably not acceptable to share	20.2	21.6	18.8
Definitely not acceptable to share	17.7	17.2	18.2

6. A health-related Government organisation, so they can send you a questionnaire to find out if you might benefit from extra care or support.

	% (TOTAL – Base = 1000)	% (SAMPLE A – Base = 500)	% (SAMPLE B – Base = 500)
Definitely acceptable to share	12.8	11.8	13.8
Probably acceptable to share	39.2	39.0	39.4
Not sure	21.2	20.6	21.8
Probably not acceptable to share	13.8	15.2	12.4
Definitely not acceptable to share	13.0	13.4	12.6

7. A specialist NHS team, to provide you with information about ways to get support for any physical or mental health issues.

	% (TOTAL – Base = 1000)	% (SAMPLE A – Base = 500)	% (SAMPLE B – Base = 500)
Definitely acceptable to share	28.1	28.2	28.0
Probably acceptable to share	44.6	44.6	44.6
Not sure	12.5	11.4	13.6
Probably not acceptable to share	7.8	8.2	7.4
Definitely not acceptable to share	7.0	7.6	6.4

8. A law firm, so they can offer to represent you in a ‘no-win, no-fee’ claim for compensation

	% (TOTAL – Base = 1000)	% (SAMPLE A – Base = 500)	% (SAMPLE B – Base = 500)
Definitely acceptable to share	2.7	3.6	1.8
Probably acceptable to share	7.8	7.2	8.4
Not sure	13.8	13.2	14.4
Probably not acceptable to share	19.7	19.4	20.0
Definitely not acceptable to share	56.0	56.6	55.4

Following a terrorist incident, people sometimes develop mental health problems as a result of their experiences. To provide help, the Government sometimes sets up a specialist NHS service to offer support to those who need it. A government organisation would be asked to help with this by putting together a confidential database of people who were present and then writing to them with information about the NHS service and a short mental health questionnaire.

Q7. To what extent do you agree or disagree with the following statement. If I was caught up in a terrorist incident, I would want the police to give my contact details to the government organisation so that they can contact me about this service.

	%
Strongly agree	21.1
Tend to agree	45.8
Neither agree nor disagree	17.5
Tend to disagree	9.7
Strongly disagree	5.9

Q8. And why do you say that? OPEN-ENDED RESPONSES

Q9. To what extent do you agree or disagree with the following statement: If I was caught up in a terrorist incident, I would want my name to be included on this database.

	%
Strongly agree	13.5
Tend to agree	36.6
Neither agree nor disagree	26.5
Tend to disagree	14.9
Strongly disagree	8.5

Q10. To what extent do you agree or disagree with the following statement: If I was caught up in a terrorist incident, I would be unhappy if my name was included on this database.

	%
Strongly agree	10.2
Tend to agree	18.5
Neither agree nor disagree	28.0
Tend to disagree	32.1
Strongly disagree	11.2

Q11A. Imagine the scenario we described occurred and the police decided to share your name and contact information with a health-related government organisation. Please say how much you agree or disagree with the following statements:

1. **I would be concerned that my details would be made public by accident**

	% (TOTAL – Base = 1000)	% (SAMPLE A – Base = 500)	% (SAMPLE B – Base = 500)
Strongly agree	27.0	30.2	23.8
Tend to agree	35.8	34.4	37.2
Neither agree nor disagree	20.7	19.8	21.6
Tend to disagree	13.8	13.4	14.2
Strongly disagree	2.7	2.2	3.2

2. **I would trust the health organisation to keep my details secure**

	% (TOTAL – Base = 1000)	% (SAMPLE A – Base = 500)	% (SAMPLE B – Base = 500)
Strongly agree	14.2	14.8	13.6
Tend to agree	39.9	36.8	43.0
Neither agree nor disagree	25.5	26.6	24.4
Tend to disagree	13.9	15.2	12.6
Strongly disagree	6.5	6.6	6.4

3. **It would be an invasion of my privacy**

	% (TOTAL – Base = 1000)	% (SAMPLE A – Base = 500)	% (SAMPLE B – Base = 500)
Strongly agree	18.6	20.8	16.4
Tend to agree	23.6	25.2	22.0
Neither agree nor disagree	30.1	29.4	30.8
Tend to disagree	23.1	20.2	26.0
Strongly disagree	4.6	4.4	4.8

4. I would be concerned about how my information might be used in the future

	% (TOTAL – Base = 1000)	% (SAMPLE A – Base = 500)	% (SAMPLE B – Base = 500)
Strongly agree	28.2	29.8	26.6
Tend to agree	40.3	38.2	42.4
Neither agree nor disagree	18.8	19.0	18.6
Tend to disagree	10.1	10.4	9.8
Strongly disagree	2.6	2.6	2.6

5. I would be concerned that my employer would be told information about me

	% (TOTAL – Base = 1000)	% (SAMPLE A – Base = 500)	% (SAMPLE B – Base = 500)
Strongly agree	11.0	11.6	10.4
Tend to agree	23.1	21.4	24.8
Neither agree nor disagree	29.5	28.2	30.8
Tend to disagree	25.4	27.4	23.4
Strongly disagree	11.0	11.4	10.6

Q11B. Imagine the scenario we described occurred and the police decided to share your name and contact information with a health-related government organisation. Please say how much you agree or disagree with the following statements:

1. I would be concerned that my details might be shared by the health organisation with other groups without my permission

	% (TOTAL – Base = 1000)	% (SAMPLE A – Base = 500)	% (SAMPLE B – Base = 500)
Strongly agree	26.1	27.2	25.0
Tend to agree	38.2	41.0	35.4
Neither agree nor disagree	21.9	18.2	25.6
Tend to disagree	11.5	11.0	12.0
Strongly disagree	2.3	2.6	2.0

2. I would want to be kept informed about how my information was being used

	% (TOTAL – Base = 1000)	% (SAMPLE A – Base = 500)	% (SAMPLE B – Base = 500)
Strongly agree	48.0	49.6	46.4
Tend to agree	35.8	35.6	36.0
Neither agree nor disagree	13.3	12.0	14.6
Tend to disagree	2.1	1.4	2.8
Strongly disagree	0.8	1.4	*

3. It wouldn't bother me at all

	% (TOTAL – Base = 1000)	% (SAMPLE A – Base = 500)	% (SAMPLE B – Base = 500)
Strongly agree	6.6	6.2	7.0
Tend to agree	22.4	21.8	23.0
Neither agree nor disagree	30.6	29.8	31.4
Tend to disagree	25.8	26.4	25.2
Strongly disagree	14.6	15.8	13.4

4. I would be reassured that a health organisation was looking out for me

	% (TOTAL – Base = 1000)	% (SAMPLE A – Base = 500)	% (SAMPLE B – Base = 500)
Strongly agree	11.8	10.8	12.8
Tend to agree	43.7	42.0	45.4
Neither agree nor disagree	28.8	28.6	29.0
Tend to disagree	10.0	12.2	7.8
Strongly disagree	5.7	6.4	5.0

5. I would be concerned that my GP might be told

	% (TOTAL – Base = 1000)	% (SAMPLE A – Base = 500)	% (SAMPLE B – Base = 500)
Strongly agree	4.5	4.4	4.6
Tend to agree	10.1	9.6	10.6
Neither agree nor disagree	28.1	29.2	27.0
Tend to disagree	36.9	35.6	38.2
Strongly disagree	20.4	21.2	19.6

Q12. Please state the highest level of education you have achieved:

	%
Left school without qualifications	2.7
Secondary education (O-level/GCSE/A-level)	41.3
Higher education (BSc, BA/higher qualification)	55.5
Prefer not to say	0.5
