# Supplementary material for: Public attitudes in England towards the sharing of personal data following a mass casualty incident: a cross-sectional study
Source: BMJ Open. 2018 May 20;8(5):e022852. doi: 10.1136/bmjopen-2018-022852 (PMC5961558; doi:10.1136/bmjopen-2018-022852)
Supplement: Supplementary file 2 [file bmjopen-2018-022852supp002.pdf]

KCL Disaster Data Protection Survey
Topline results

- Results are based on interviews with 1000 members of the public aged 18-65 across England. Interviews were conducted between 9 March 2017 and 15 March 2017.
- All interviews were conducted via Ipsos MORI's online panel.
- The survey data is unweighted but based on quotas on age, gender, region and working status to reflect the profile of adults aged 18-65 in England, with up to 10% flexibility. Weights are available in the SPSS file for the whole sample, Sample A and Sample B.
- Results are based on all respondents (1000) unless otherwise stated. Bases specified are unweighted.
- Where percentages do not sum to 100, this may be due to respondents being able to select multiple responses, computer rounding or the exclusion of 'don't know'/ not stated.
- An asterisk (*) represents a value of less than half or one percent, but greater than zero.
- The response rate for this survey was 10% (1000 responses from 10004 people that the link was emailed to).
- 25 participants ended the survey without completing it (and were not included in the data); 26 people were excluded from the data due to being identified as 'speeding' or straightlining'; i.e. completing the survey too quickly to have given genuine, considered answers, or providing identical answers to five or more consecutive questions where this was possible.

Q1. Please select your date of birth (recoded into age categories):

	%
18-24	12
25-34	23
35-44	21
45-54	23
55-65	21

Q2. Please state the gender you identify yourself with:

	%
Male	49
Female	51

Q3. In which of the following regions do you live?

	%
North East	5
North West	14
Yorkshire and Humberside	10
West Midlands	10
East Midlands	9
East of England	9
South West	10
South East	17
London	18

Q4. Which of the following best describes your employment status?

	%
Working – full time (30 or more hours a week)	50
Working – part-time (less than 30 hours a week)	15
Self-employed	7
Unemployed – looking for a job	5
Unemployed – not looking for a job/long-term sick or disabled/Housewife/husband/Full-time carer	11
Retired	8
Pupil/Student/In full-time education	5
**NET: Working**	72
**NET: Not working**	28

We are interested in people's views about sharing information following a major incident such as a terrorist attack. The researchers have no reason to think that an attack is going to happen. This research is one of a number of studies that academics at King's College London are conducting on how people might react to a range of different issues.

SAMPLE A:
Imagine that you are on holiday in another country and witness a terrorist shooting. Some people are badly injured but you are not harmed and neither is anyone you know well. The British government arranges for you to be flown home, along with other British nationals who were in the area. When you arrive back in Britain, a police officer at the airport records your name, address, phone number and email address. The following questions ask about things that might happen next.

SAMPLE B:
Imagine that you are on holiday in another country and a place you visit is discovered by police to be contaminated by radioactive material. The police believe this is linked to a terrorist group. Some people are badly injured but you are not harmed and neither is anyone you know well. The British government arranges for you to be flown home, along with other British nationals who were in the area. When you arrive back in Britain, a police officer at the airport records your name, address, phone number and email address. The following questions ask about things that might happen next.

Q5. (TOTAL) Following an incident like this, various groups or individuals might ask the police to tell them who was caught up in the incident. Please say whether you think that the police are or are not currently able to share your name and contact details with these groups or people following an incident like this, without checking with you first.

1. **Your GP, so they can check how you are doing**

	% (TOTAL – Base = 1000)	% (SAMPLE A – Base = 500)	% (SAMPLE B – Base = 500)
Definitely able to share	18	14	22
Probably able to share	36	37	35
Not sure	21	21	20
Probably not able to share	15	18	13
Definitely not able to share	10	10	10

2. **Your travel insurance company, so they can offer you practical and financial support**

	% (TOTAL – Base = 1000)	% (SAMPLE A – Base = 500)	% (SAMPLE B – Base = 500)
Definitely able to share	8	8	9
Probably able to share	24	25	23
Not sure	27	27	28
Probably not able to share	24	26	22

Definitely not able to share	17	16	19

3. A team of medical researchers from a university, so they can invite you to take part in a study to improve the way future incidents are dealt with

	% (TOTAL – Base = 1000)	% (SAMPLE A – Base = 500)	% (SAMPLE B – Base = 500)
Definitely able to share	5	4	6
Probably able to share	14	14	13
Not sure	22	22	23
Probably not able to share	31	31	30
Definitely not able to share	28	29	28

4. A journalist, so they can write a news article about the incident

	% (TOTAL – Base = 1000)	% (SAMPLE A – Base = 500)	% (SAMPLE B – Base = 500)
Definitely able to share	3	3	2
Probably able to share	5	4	7
Not sure	13	13	13
Probably not able to share	20	22	19
Definitely not able to share	59	58	59

5. A charity, such as the British Red Cross, so they can offer you support

	% (TOTAL – Base = 1000)	% (SAMPLE A – Base = 500)	% (SAMPLE B – Base = 500)
Definitely able to share	6	5	7
Probably able to share	18	20	16
Not sure	28	29	28
Probably not able to share	29	28	30
Definitely not able to share	19	18	20

6. A health-related Government organisation, so they can send you a questionnaire to find out if you might benefit from extra care or support.

	% (TOTAL – Base = 1000)	% (SAMPLE A – Base = 500)	% (SAMPLE B – Base = 500)
Definitely able to share	10	8	13
Probably able to share	36	33	39
Not sure	27	29	25
Probably not able to share	16	18	15
Definitely not able to share	11	13	9

7. A specialist NHS team, to provide you with information about ways to get support for any physical or mental health issues.

	% (TOTAL – Base = 1000)	% (SAMPLE A – Base = 500)	% (SAMPLE B – Base = 500)
Definitely able to share	18	14	22
Probably able to share	43	41	45
Not sure	21	25	17
Probably not able to share	11	14	9
Definitely not able to share	7	7	7

8. **A law firm, so they can offer to represent you in a 'no-win, no-fee' claim for compensation**

	% (TOTAL – Base = 1000)	% (SAMPLE A – Base = 500)	% (SAMPLE B – Base = 500)
Definitely able to share	4	3	4
Probably able to share	9	9	9
Not sure	18	17	20
Probably not able to share	25	23	27
Definitely not able to share	44	48	41

Q6. Following an incident like this, various groups or individuals might ask the police to tell them who was caught up in the incident. For each of the following groups, please say whether you think that is acceptable or not acceptable for the police to share your name and contact details with these groups or people following an incident like this, without checking with you first;

1. **Your GP, so they can check how you are doing**

	% (TOTAL – Base = 1000)	% (SAMPLE A – Base = 500)	% (SAMPLE B – Base = 500)
Definitely acceptable to share	30	26	33
Probably acceptable to share	39	38	40
Not sure	12	14	10
Probably not acceptable to share	10	11	9
Definitely not acceptable to share	9	10	8

2. **Your travel insurance company, so they can offer you practical and financial support**

	% (TOTAL – Base = 1000)	% (SAMPLE A – Base = 500)	% (SAMPLE B – Base = 500)
Definitely acceptable to share	11	9	13
Probably acceptable to share	27	30	25
Not sure	23	23	24
Probably not acceptable to share	16	16	16
Definitely not acceptable to share	22	22	22

3. **A team of medical researchers from a university, so they can invite you to take part in a study to improve the way future incidents are dealt with**

	% (TOTAL – Base = 1000)	% (SAMPLE A – Base = 500)	% (SAMPLE B – Base = 500)
Definitely acceptable to share	6	5	8
Probably acceptable to share	20	19	22
Not sure	21	22	21
Probably not acceptable to share	23	23	23
Definitely not acceptable to share	29	31	27

4. **A journalist, so they can write a news article about the incident**

	% (TOTAL – Base = 1000)	% (SAMPLE A – Base = 500)	% (SAMPLE B – Base = 500)
Definitely acceptable to share	2	1	2
Probably acceptable to share	4	4	4
Not sure	8	10	7
Probably not acceptable to share	14	13	14
Definitely not acceptable to share	72	72	73

5. A charity, such as the British Red Cross, so they can offer you support

	% (TOTAL – Base = 1000)	% (SAMPLE A – Base = 500)	% (SAMPLE B – Base = 500)
Definitely acceptable to share	8	7	8
Probably acceptable to share	27	27	27
Not sure	25	27	24
Probably not acceptable to share	20	19	20
Definitely not acceptable to share	20	20	20

6. A health-related Government organisation, so they can send you a questionnaire to find out if you might benefit from extra care or support.

	% (TOTAL – Base = 1000)	% (SAMPLE A – Base = 500)	% (SAMPLE B – Base = 500)
Definitely acceptable to share	13	10	17
Probably acceptable to share	36	35	37
Not sure	23	25	20
Probably not acceptable to share	15	16	15
Definitely not acceptable to share	13	14	12

7. A specialist NHS team, to provide you with information about ways to get support for any physical or mental health issues.

	% (TOTAL – Base = 1000)	% (SAMPLE A – Base = 500)	% (SAMPLE B – Base = 500)
Definitely acceptable to share	27	23	31
Probably acceptable to share	44	43	45
Not sure	14	16	12
Probably not acceptable to share	8	9	7
Definitely not acceptable to share	7	8	6

8. A law firm, so they can offer to represent you in a 'no-win, no-fee' claim for compensation

	% (TOTAL – Base = 1000)	% (SAMPLE A – Base = 500)	% (SAMPLE B – Base = 500)
Definitely acceptable to share	3	3	4
Probably acceptable to share	8	8	9
Not sure	13	14	11
Probably not acceptable to share	16	14	18
Definitely not acceptable to share	60	61	59

Following a terrorist incident, people sometimes develop mental health problems as a result of their experiences. To provide help, the Government sometimes sets up a specialist NHS service to offer support to those who need it. A government organisation would be asked to help with this by putting together a confidential database of people who were present and then writing to them with information about the NHS service and a short mental health questionnaire.

Q7. To what extent do you agree or disagree with the following statement. If I was caught up in a terrorist incident, I would want the police to give my contact details to the government organisation so that they can contact me about this service.

	%
Strongly agree	21
Tend to agree	45
Neither agree nor disagree	18
Tend to disagree	12
Strongly disagree	4

Q8. And why do you say that? OPEN-ENDED RESPONSES

Q9. To what extent do you agree or disagree with the following statement: If I was caught up in a terrorist incident, I would want my name to be included on this database.

	%
Strongly agree	14
Tend to agree	39
Neither agree nor disagree	24
Tend to disagree	16
Strongly disagree	7

Q10. To what extent do you agree or disagree with the following statement: If I was caught up in a terrorist incident, I would be unhappy if my name was included on this database.

	%
Strongly agree	9
Tend to agree	18
Neither agree nor disagree	31
Tend to disagree	32
Strongly disagree	10

Q11A. Imagine the scenario we described occurred and the police decided to share your name and contact information with a health-related government organisation. Please say how much you agree or disagree with the following statements:

1. **I would be concerned that my details would be made public by accident**

	% (TOTAL – Base = 1000)	% (SAMPLE A – Base = 500)	% (SAMPLE B – Base = 500)
Strongly agree	26	28	24
Tend to agree	37	38	35
Neither agree nor disagree	22	20	23
Tend to disagree	14	13	14
Strongly disagree	2	2	3

2. **I would trust the health organisation to keep my details secure**

	% (TOTAL – Base = 1000)	% (SAMPLE A – Base = 500)	% (SAMPLE B – Base = 500)
Strongly agree	17	16	19
Tend to agree	38	36	39
Neither agree nor disagree	26	27	25
Tend to disagree	15	18	13
Strongly disagree	5	4	5

3. **It would be an invasion of my privacy**

	% (TOTAL – Base = 1000)	% (SAMPLE A – Base = 500)	% (SAMPLE B – Base = 500)
Strongly agree	16	17	15
Tend to agree	25	26	23
Neither agree nor disagree	29	32	27
Tend to disagree	24	21	28
Strongly disagree	5	4	6

4. **I would be concerned about how my information might be used in the future**

	% (TOTAL – Base = 1000)	% (SAMPLE A – Base = 500)	% (SAMPLE B – Base = 500)
Strongly agree	27	30	24
Tend to agree	41	41	40
Neither agree nor disagree	19	16	22
Tend to disagree	12	11	12
Strongly disagree	2	2	2

5. **I would be concerned that my employer would be told information about me**

	% (TOTAL – Base = 1000)	% (SAMPLE A – Base = 500)	% (SAMPLE B – Base = 500)
Strongly agree	12	12	11
Tend to agree	23	25	21
Neither agree nor disagree	28	28	29
Tend to disagree	27	25	29
Strongly disagree	11	11	11

Q11B. Imagine the scenario we described occurred and the police decided to share your name and contact information with a health-related government organisation. Please say how much you agree or disagree with the following statements:

1. **I would be concerned that my details might be shared by the health organisation with other groups without my permission**

	% (TOTAL – Base = 1000)	% (SAMPLE A – Base = 500)	% (SAMPLE B – Base = 500)
Strongly agree	25	27	22
Tend to agree	38	36	41
Neither agree nor disagree	23	22	23
Tend to disagree	11	11	12
Strongly disagree	3	4	3

2. **I would want to be kept informed about how my information was being used**

	% (TOTAL – Base = 1000)	% (SAMPLE A – Base = 500)	% (SAMPLE B – Base = 500)
Strongly agree	52	52	51
Tend to agree	34	32	35
Neither agree nor disagree	11	11	11
Tend to disagree	3	3	2
Strongly disagree	1	1	*

3. **It wouldn't bother me at all**

	% (TOTAL – Base = 1000)	% (SAMPLE A – Base = 500)	% (SAMPLE B – Base = 500)
Strongly agree	6	5	7
Tend to agree	20	19	21
Neither agree nor disagree	33	32	35
Tend to disagree	26	28	24
Strongly disagree	15	16	13

4. I would be reassured that a health organisation was looking out for me

	% (TOTAL – Base = 1000)	% (SAMPLE A – Base = 500)	% (SAMPLE B – Base = 500)
Strongly agree	14	11	17
Tend to agree	42	43	40
Neither agree nor disagree	32	32	31
Tend to disagree	9	10	8
Strongly disagree	4	4	4

5. I would be concerned that my GP might be told

	% (TOTAL – Base = 1000)	% (SAMPLE A – Base = 500)	% (SAMPLE B – Base = 500)
Strongly agree	4	4	4
Tend to agree	9	11	7
Neither agree nor disagree	29	30	28
Tend to disagree	40	38	41
Strongly disagree	18	17	19

Q12. Please state the highest level of education you have achieved:

	%
Left school without qualifications	3
Secondary education (O-level/GCSE/A-level)	42
Higher education (BSc, BA/higher qualification)	54
Prefer not to say	1
