# Supplementary material for: Public attitudes in England towards the sharing of personal data following a mass casualty incident: a cross-sectional study
Source: BMJ Open. 2018 May 20;8(5):e022852. doi: 10.1136/bmjopen-2018-022852 (PMC5961558; doi:10.1136/bmjopen-2018-022852)
Supplement: Author's manuscript [file bmjopen-2018-022852.draft_revisions.pdf]

BMJ Open is committed to open peer review. As part of this commitment we make the peer review history of every article we publish publicly available.

When an article is published we post the peer reviewers' comments and the authors' responses online. We also post the versions of the paper that were used during peer review. These are the versions that the peer review comments apply to.

The versions of the paper that follow are the versions that were submitted during the peer review process. They are not the versions of record or the final published versions. They should not be cited or distributed as the published version of this manuscript.

BMJ Open is an open access journal and the full, final, typeset and author-corrected version of record of the manuscript is available on our site with no access controls, subscription charges or pay-per-view fees (http://bmjopen.bmj.com).

If you have any questions on BMJ Open's open peer review process please email info.bmjopen@bmj.com

BMJ Open

Public attitudes towards the sharing of personal data following a mass casualty incident: A cross-sectional study

Journal:	*BMJ Open*
Manuscript ID	bmjopen-2018-022852
Article Type:	Research
Date Submitted by the Author:	12-Mar-2018

Complete List of Authors:	Rubin, GJ; King's College London, Psychological Medicine<br>Webster, Rebecca; King's College London, Psychological Medicine<br>Rubin, Antonia<br>Amlot, Richard; Public Health England, Behavioural Science Emergency Response Department Science and Technology<br>Grey, N; Sussex Partnership NHS Foundation Trust<br>greenberg, neil; Kings College London, Kings Centre for Military Health Research

Keywords:	Data sharing, Mass casualty incident, Public attitudes, Terrorism, Shooting, Radiation

SCHOLARONE™ Manuscripts

For peer review only

Public attitudes towards the sharing of personal data following a mass casualty incident: A cross-sectional study

G James Rubin, Rebecca Webster, Antonia N Rubin, Richard Amlôt, Nick Grey, Neil Greenberg

G James Rubin, Reader in the Psychology of Emerging Health Risks, Department of Psychological Medicine, King's College London

Rebecca Webster, Postdoctoral researcher, Department of Psychological Medicine, King's College London

Antonia N Rubin, Lawyer, Tonbridge, Kent.

Richard Amlôt, Scientific Programme Leader, Behavioural Science Emergency Response Department Science and Technology (ERD S&T), Health Protection Directorate, Porton Down, Salisbury, Public Health England

Nick Grey, Consultant Clinical Psychologist, Aldrington House, Sussex Partnership NHS Foundation Trust, 35 New Church Road, Hove, BN3 4AG, and University of Sussex

Neil Greenberg, Professor of Defence Mental Health, Department of Psychological Medicine, King's College London

Correspondence to: Gideon.rubin@kcl.ac.uk

Word count: 3864

For peer review only

Abstract

Objectives: To assess public attitudes towards data sharing to facilitate a mental health screening programme for people caught up in a mass casualty incident.

Design: Two, identical, cross-sectional, on-line surveys, using quotas to ensure demographic representativeness of 18 to 65 year olds in England. Participants were randomly allocated to consider a scenario in which they witness a terrorism-related radiation incident or mass shooting, after which a police officer records their contact details.

Setting: Participants were drawn from an on-line panel maintained by a market research company. Surveys were conducted before and immediately after a series of terrorist attacks and a large tower block fire occurred in England.

Participants: One thousand people aged 18 to 65 years participated in each survey.

Main outcome measures: Three questions asking participants if it would acceptable for police to share their contact details, without asking first, with “a health-related government organisation, so they can send you a questionnaire to find out if you might benefit from extra care or support,” “a specialist NHS team, to provide you with information about ways to get support for any physical or mental health issues,” and “your GP, so they can check how you are doing.”

Results: A minority of participants reported that it would be unacceptable for their details to be shared with the government organisation (n=550, 27.5%), the NHS (n=298, 14.9%) and their GP (n=347, 17.4%). There was a small, but significant increase in acceptability for the radiation incident compared to the mass shooting. No differences were observed between the pre-incidents and post-incidents surveys.

Conclusions: Although most people believe it is acceptable for their details to be shared in order to facilitate a mental health response to a major incident, care must be taken to communicate with those affected about how their information will be used.

Key words: data sharing, mass casualty incident, public attitudes, terrorism, shooting, radiation

For peer review only - http://bmjopen.bmj.com/site/about/guidelines.xhtml

Strengths and limitations

- The survey sample was demographically representative of 18 to 65 year olds in England.
- The survey was conducted again after the recent terrorist incidents and the Grenfell tower fire as we felt that the scenarios may have become more salient for respondents, providing a better test of attitudes towards data sharing in such contexts.
- Although demographically representative, our sample volunteered to answer internet surveys and may be more accepting of data sharing than those who did not volunteer.
- The scenarios used in our surveys were hypothetical. Being directly involved in a major incident may alter someone's views about the desirability of data sharing.
- Participants were asked to consider that neither they, nor anyone they knew well, had been harmed in the described scenarios. This may have reduced the perceived need to participate in a psychological screening programme and hence the perceived acceptability of data sharing.

What this paper adds

What is already known on this subject:

- Recent polling suggests that public attitudes towards data sharing are shaped by who is doing the sharing, what it is for, and whether safeguards are in place.
- Concerns about data protection legislation have previously hampered data sharing between official agencies in the aftermath of terrorist attacks.
- To our knowledge there has been no research on the public acceptability of data sharing in the aftermath of a major public health incident, disaster or terrorist attack.

What this study adds:

- While most members of the public support the sharing of data between the police and health services in order to facilitate public health measures, a minority do not believe this is acceptable.
- The absence of a clear consensus reinforces the view that agencies should consider the benefits and risks of sharing data with organisations that seek to protect their mental health.

For peer review only - http://bmjopen.bmj.com/site/about/guidelines.xhtml

Introduction

Following a disaster, terrorist attack or other mass casualty incident, rates of mental distress and disorder among survivors, their relatives, eyewitnesses and first responders can be substantial (1). Although evidence-based interventions are available to treat established mental health disorder, failure to seek professional help for mental illness is common as a result of stigma and other barriers to care (2, 3). Because of this, in their guidance on the detection and treatment of post-traumatic stress disorder, the UK National Institute for Health and Clinical Excellence recommends that, "screening of all [affected] individuals should be considered by the authorities responsible for developing the local disaster plan."(4) Within the UK the first use of a 'screen and treat' approach occurred following the 7 July 2005 bombings in London. A central team used a range of methods to contact as many people as possible who had been caught up in the incident. Those contacted were asked to complete a short mental health questionnaire, invited to have a more detailed assessment if need-be, and referred for treatment where required (5). Although the process resulted in many patients receiving treatment who might not otherwise have done so, difficulties were encountered in assembling a comprehensive list of people to be screened. Sharing of information between agencies was hampered by a cautious interpretation of legislation designed to safeguard personal data (6). As a result, only 910 people were contacted out of an estimated 4,000 who were exposed to the attacks. Within the UK, greater clarity about the flexibility that responders have to share information was subsequently provided (6). This guidance notes that where information is not "sensitive" (i.e. related to ethnicity, political opinion, religious belief, trade union membership, health, sexual life or criminal activity) then it is acceptable for names and contact details to be shared between official agencies to facilitate the provision of care to those affected.

In June 2015, 38 people were killed by a terrorist in Sousse, Tunisia. Thirty of those killed were British holidaymakers. Given the large number of British tourists who witnessed the attacks and felt their own lives to be in danger, a psychological health screening programme was again set up and subsequently widened to include victims of terrorist attacks in Brussels and Paris. Public Health England was given the task of setting up a registry of all English nationals who were directly affected by the attacks, something which might also be considered in the future for other forms of disaster(7). Once again, however, obtaining the contact details for many of those affected was not possible. Although the police collected contact details from holiday makers as they returned to England, legal advice prevented them from sharing this information. Difficulty in sharing information between agencies often hampers response efforts following a major incident. While it is sometimes possible to find more indirect routes to contact people, these approaches are often less than ideal. For example, while it might be possible for the police to send out information to people on behalf of a screening service or to ask for permission before sharing data, in practice, such strategies can be time consuming to agree, often result in a poorer response rate and prevent any single agency from

For peer review only - http://bmjopen.bmj.com/site/about/guidelines.xhtml

combining overlapping lists of names held by different groups into a single, consolidated list of those affected.

Clearly, uncertainties still exist about when it is appropriate to share information following a major incident. Changes to legislation in 2018 will create stiffer penalties for organisations found to be in breach of data protection law and are likely to create an even more cautious approach to data sharing. But how members of the public themselves view this matter is unclear. Recent polling suggests that public attitudes towards data sharing are shaped by who is doing the sharing, what for, and whether safeguards are in place (8). Data sharing for the public good is generally viewed positively. For example, only 9% of respondents to one large UK survey felt that it would be an invasion of privacy if details held about them on a cancer registry were used to invite them to participate in a study conducted by a university medical school (9). Where a personal benefit is possible, people also tend to be more willing for information to be shared (10). To our knowledge there has been no research on the public acceptability of data sharing in the context of a major public health incident, disaster or terrorist attack.

In this study, we used an on-line quota survey of a sample of adults in England designed to reflect the known population profile, to identify public attitudes towards the sharing of personal information following a mass casualty incident and the setting up of a register of affected people. We tested whether attitudes might differ depending on whether the incident resulted primarily in a mental or physical health risk for those involved in the incident. Soon after we conducted our survey, four terrorist attacks and a tower block fire occurred in England killing over a hundred people. Given the intense media attention about the need to provide support to victims of these incidents, we felt that the scenarios described in our survey may have become more salient for respondents, providing a better test of attitudes towards data sharing in such contexts. We therefore repeated the survey with a new sample of respondents.

Method

Design

We commissioned the market research organisation Ipsos MORI to conduct two on-line surveys of 1,000 adult (18 to 65) residents of England. Data collection for the initial, pre-incidents survey took place between 9 and 15 March 2017. Four terrorist attacks then occurred in England between 22 March and 19 June, killing 34, and a tower block fire occurred on 14 June, killing 71. Data collection for our second, post-incidents survey took place between 3 and 10 July 2017. The procedures and questionnaires for both surveys were identical. The study was approved by the Psychiatry, Nursing and Midwifery Research Ethics Committee at King's College London (ref: HR-16/17-3814).

For peer review only - http://bmjopen.bmj.com/site/about/guidelines.xhtml

Participants

Ipsos MORI recruited participants from an existing panel of people willing to take part in internet surveys (approximate N=160,000). Panel participants typically receive points for every survey they complete: for our surveys, participants received points equivalent to 30 pence. Quotas based on participant age and gender (interlocked), location, and working status were used to ensure that the sample was demographically representative of adults aged 18-65 in England, according to data from the National Readership Survey (11). We excluded adults aged over 65 on the basis that older adults who are members of an internet survey panel may not be representative of the general population of older adults (12). We intended to recruit 1,000 participants for each survey to provide us with a maximum sample error of about plus or minus 3%.

Scenarios

Ipsos MORI emailed a link to the survey to potential participants. After providing informed consent and clicking through to begin the survey, participants received questions about one of two hypothetical scenarios. Which scenario they received was decided by the survey software, based on which scenario had the lowest number of completed responses at that time. We based the scenarios on an example given in HM Government's 'Data Protection and Sharing – Guidance for Emergency Planners and Responders' (6) (case study 5). The two versions represented an incident that might primarily pose a mental health threat (witnessing shooting) or a physical health threat (exposure to radiation). In the first version, participants were asked to, "imagine that you are on holiday in another country and witness a terrorist shooting. Some people are badly injured but you are not harmed and neither is anyone you know well." The second version asked them to, "imagine that you are on holiday in another country and a place you visit is discovered by police to be contaminated by radioactive material. The police believe this is linked to a terrorist group. Some people are badly injured but you are not harmed and neither is anyone you know well." Participants in both versions were then told that "the British government arranges for you to be flown home, along with other British nationals who were in the area. When you arrive back in Britain, a police officer at the airport records your name, address, phone number and email address. The following questions ask about things that might happen next."

Questionnaire

The supplementary materials show the full text for all items. We first presented participants with eight groups who might ask the police to share information with them following the incident, together with their reasons for this. In each case we asked whether the participant thought the police currently could share information with this group without asking the participant first, and whether the participant thought it was acceptable for the police to share their information with the group.

For peer review only - http://bmjopen.bmj.com/site/about/guidelines.xhtml

We next provided information about the rationale and process for a mental health screen and treat programme. We described it as a specialist NHS service set up to offer support and treatment to those who need it and explained that a government organisation would support the service by putting together a confidential database of those affected and writing to them with a screening questionnaire. We asked participants to state their level of agreement with the statements that, "If I was caught up in a terrorist incident, I would want the police to give my contact details to the government organisation so that they can contact me about this service;" "I would want my name to be included on this database;" and "I would be unhappy if my name was included on this database."

Participants were then asked to reconsider the scenario they had been presented with and told that the police had decided to share their name and contact details with a health-related government organisation. They were asked to state their agreement with 10 statements concerning their views about this (e.g. "it would be an invasion of my privacy").

We asked all participants about their age, gender, working status, where in England they lived and their highest educational qualification.

Analysis

The five response options for each set of questions were collapsed into three categories to assist in interpretation (see Tables 1 to 5). We calculated the proportion of respondents endorsing each response option and assessed the difference in responses between the pre- and post-incidents surveys and between the two scenarios using chi-square tests.

Patient Involvement

No patients or members of the public were involved in the design, recruitment or conduct of the study. Due to anonymity, results cannot be disseminated to study participants unless they specifically request them from the researcher.

Results

The topline results provided by Ipsos MORI for both surveys can be found in supplementary file 1 and supplementary file 2.

Response rates and demographics

Response rates to the invitation emails were 10% (pre-incident) and 6% (post-incident). These are normal rates for surveys of this nature. In each case, a small proportion of respondents (45 in total) were excluded from the data for completing the surveys suspiciously quickly or giving illogically identical answers to multiple consecutive questions. Demographics are shown in Table 1.

For peer review only - http://bmjopen.bmj.com/site/about/guidelines.xhtml

Perceived ability and acceptability of the police sharing data with other agencies

Table 2 presents data on the perceived ability of the police to share personal data with other agencies. The option that was most commonly believed to be within the police's current powers was sharing data with "a specialist NHS team, to provide you with information about ways to get support for any physical or mental health issues" (endorsed by 60.8%). 53.9% of respondents believed that police could share data with "your GP, so they can check how you are doing," while 45.9% believed that data could be shared with "a health-related government organisation, so they can send you a questionnaire to find out if you might benefit from extra care or support." No differences were found between the pre- and post-incident surveys. Significantly more people believed that data could be shared following a radiation incident compared to a shooting incident with: your GP (58.1% vs 49.7%, respectively); a team of university medical researchers (20.1% vs 16.1%); a health-related government organisation (50.9% vs 40.9%); and a specialist NHS team (65.3% vs 56.3%).

Table 3 presents data on the acceptability of the police sharing personal data. The option that was most commonly seen as being acceptable was sharing data with a specialist NHS team (endorsed by 71.9%). 70.1% of respondents believed it was acceptable for the police to share data with their GP, while 50.6% believed it was acceptable for data to be shared with a health-related government organisation. No differences were found between the pre- and post-incident surveys. Significantly more people believed it would be acceptable to share their data following a radiation incident compared to a shooting incident with: their GP (72.8% vs 67.4%, respectively); a team of university medical researchers (27.6% vs 21.6%); and a health-related government organisation (53.5% vs 47.8%).

Attitudes towards data sharing to enable a screen and treat programme or database

Table 4 presents the attitudes of participants towards a screen and treat programme and confidential database. Although 66.6% of respondents would want the police to share their contact details to allow them to be contacted about screening, 15.8% did not. 51.6% reported wanting their name to appear on the database, while 27.9% reported that they would be unhappy if it was included on the database. No differences were found between the pre- and post-incident surveys. Because these questions asked about "a terrorist incident" in general, we did not test for differences between the shooting and radiation scenarios.

Table 5 presents the broader attitudes of participants towards data-sharing in the context of the radiation and shooting scenarios. Concerns endorsed by more than 50% of respondents about the police sharing their information were that: my details would be made public by accident (62.6%); I would be concerned about how my information might be used in the future (67.8%); and I would be concerned that my details might be shared by the health organisation with other groups without my

For peer review only - http://bmjopen.bmj.com/site/about/guidelines.xhtml

permission (63.5%). Most (84.8%) participants reported wanting to be kept informed about how their information was being used. More positively, 55.4% would be “reassured that a health organisation was looking out for me” and 54.4% would trust the health organisation to keep their details secure. No differences were found between and pre- and post-incidents surveys. Significantly more people in the radiation than the shooting scenario reported that the sharing of information would not bother them at all (29.4% vs 26.2%), that they would be reassured that a health organisation was looking out for them (57.5% vs 53.2%) and that they would trust the health organisation to keep their details secure (57.2% vs 51.5%). Significantly fewer people in the radiation scenario reported that the sharing would be an invasion of their privacy (38.6% vs 44.6%).

For peer review only

For peer review only - http://bmjopen.bmj.com/site/about/guidelines.xhtml

Discussion

Our data suggest that not only do the majority of people believe that it is acceptable for the police to share their details with health agencies in the aftermath of a major incident, but that most people believe that the police are currently able to do this under existing legislation. This belief is not simply due to a broader misunderstanding among the public about their data protection rights: only 8% believe that the police are able to share data with journalists, for example. Instead, in line with findings reported by others (10), it appears that where a direct benefit to the individual might result from sharing information with a trusted source such as a GP or NHS service, the public have a greater acceptability of it, and an expectation that it will occur. However, such views were not unanimous among our participants. It was notable that 15% of our sample did not think it acceptable for their details to be shared with an NHS service following a major incident, 17% did not think it acceptable for their details to be shared with their GP, and 27% would be unhappy for their name to be included on a confidential database of those affected. These figures must be taken into consideration by responding agencies. Given that it is impossible to know in advance who is or is not willing for their data to be shared, not sharing data without prior permission to protect the interests of this minority may be a justifiable position in some cases.

Our data provide some indications as to why people may be concerned about data sharing following an incident. In particular, the risk of accidental or deliberate sharing of information to a third party was noted as a concern by many. If data sharing or the setting up of a database is to be considered following any future incident, a robust policy around further sharing should be developed and explained to those affected. Keeping people regularly updated on how their data are being used is also important. More generally, the nature of the incident itself is a determinant of the acceptability of data sharing and the use of a database. Participants asked to imagine a scenario that might pose a long-term physical health risk to themselves (possible exposure to radiation) were more likely to have a positive view of data sharing than those asked to consider a risk to their mental health (witnessing a shooting). Whether this was because participants found it harder to envisage that their mental health might be affected in the scenarios that we used, or whether this reflects the broader stigma associated with mental illness is unclear. In either case, additional care may be required when communicating with the public about data sharing that is primarily intended to support a mental health response. It is possible that ongoing public campaigns to improve the understanding of mental ill-health may impact upon these differences over time.

Limitations

Several limitations with our data need to be considered. First, we restricted our sample to people aged 18 to 65. We are unable to extrapolate from our data to those older or younger than this. Second, although our sample was demographically representative of 18 to 65 year olds in England, it may not

For peer review only - http://bmjopen.bmj.com/site/about/guidelines.xhtml

have been psychologically representative of this population. In particular, it seems plausible that people who volunteer to answer internet surveys may be more accepting of data sharing than those who do not volunteer. If anything, our estimate of the number of people expressing reticence about data sharing is therefore likely to be an underestimate. Third, the scenarios used in our surveys were hypothetical. We hoped that repeating our survey soon after a series of terrorist attacks and a major disaster would make the issues involved more salient for participants. Doing this did not alter our results. Nonetheless, it is possible that being directly involved in a major incident would alter someone's views about the desirability of data sharing. Fourth, for ethical reasons, we asked participants to consider that neither they, nor anyone they knew well, had been harmed in the scenarios that we described. This may have reduced the perceived need to participate in a psychological screening programme and hence the perceived acceptability of data sharing. People who are injured, bereaved or otherwise harmed in an incident may be more willing for their data to be shared. Finally, we have no information on our participants' previous experiences of data sharing or data protection breaches. Prior experience of breaches, from whatever source or agency, may make people more reluctant to consider future data sharing. This may be relevant for the minority of people in our sample who were reluctant to share data. It would also reinforce the need for clear information and reassurance about data handling.

Conclusion

Disagreement often exists between officials and agencies about the appropriateness of, and best mechanisms for, data sharing in the aftermath of a major incident. These disagreements are also apparent among members of the public: while most support the sharing of data between the police and health services in order to facilitate public health measures, this is by no means a unanimous view. The absence of a clear consensus reinforces the view that agencies should consider the benefits and risks of sharing data with organisations that seek to protect their mental health. In order to assuage public concerns, agencies who hold such data should communicate to the public their policies for securely holding the data and for updating people on how the data will be used.

Author contributions

GJR developed the idea for the survey and the survey materials in conjunction with ANR, RA, NG and NG, Ipsos MORI carried out the data collection. RW carried out the data analysis. GJR drafted the manuscript and revised the manuscript with comments from all authors. GJR accepts full responsibility for the work, had access to the data and controlled the decision to publish.

Financial disclosure statement

For peer review only - http://bmjopen.bmj.com/site/about/guidelines.xhtml

The research was funded by the National Institute for Health Research Health Protection Research Unit (NIHR HPRU) in Emergency Preparedness and Response at King's College London in partnership with Public Health England (PHE), and by the NIHR HPRU in Evaluation of Interventions at the University of Bristol in partnership with PHE. The views expressed are those of the author(s) and not necessarily those of the NHS, the NIHR, the Department of Health or Public Health England.

Copyright statement

The Corresponding Author has the right to grant on behalf of all authors and does grant on behalf of all authors, a worldwide licence to the Publishers and its licensees in perpetuity, in all forms, formats and media (whether known now or created in the future), to i) publish, reproduce, distribute, display and store the Contribution, ii) translate the Contribution into other languages, create adaptations, reprints, include within collections and create summaries, extracts and/or, abstracts of the Contribution, iii) create any other derivative work(s) based on the Contribution, iv) to exploit all subsidiary rights in the Contribution, v) the inclusion of electronic links from the Contribution to third party material where-ever it may be located; and, vi) licence any third party to do any or all of the above.

Declaration of competing interests

Competing interests: All authors have completed the ICMJE uniform disclosure form at www.icmje.org/coi_disclosure.pdf and declare: support from the NIHR HPRUs in Emergency Preparedness and Response and in Evaluation of Interventions for the submitted work; no financial relationships with any organisations that might have an interest in the submitted work in the previous three years; no other relationships or activities that could appear to have influenced the submitted work.

Full access to data

All authors had full access to all of the data in the study and can take responsibility for the integrity of the data and the accuracy of the data analysis.

Ethics approval

The study was approved by the Psychiatry, Nursing and Midwifery Research Ethics Committee at King's College London (ref: HR-16/17-3814). All participants provided informed consent before starting the surveys.

Transparency declaration

For peer review only - http://bmjopen.bmj.com/site/about/guidelines.xhtml

The lead author confirms that this manuscript is an honest, accurate and transparent account of the study being reported. No important aspects of the study have been omitted and any discrepancies from the study as planned have been explained.

Data sharing

No additional data available.

For peer review only

For peer review only - http://bmjopen.bmj.com/site/about/guidelines.xhtml

References

1. Whalley MG, Brewin CR. Mental health following terrorist attacks. British Journal of Psychiatry. 2007;190(FEB.):94-6.

2. Stuber J, Galea S, Boscarino JA, Schlesinger M. Was there unmet mental health need after the September 11, 2001 terrorist attacks? Social Psychiatry and Psychiatric Epidemiology. 2006;41(3):230-40.

3. Borschmann R, Greenberg N, Jones N, Henderson RC. Campaigns to reduce mental illness stigma in Europe: A scoping review. Die Psychiatrie. 2014;11(1):43-50.

4. Excellence NIfHaC. Post-traumatic stress disorder: management. Manchester: National Institute for Health and Clinical Excellence; 2005.

5. Brewin CR, Fuchkan N, Huntley Z, Robertson M, Thompson M, Scragg P, et al. Outreach and screening following the 2005 London bombings: Usage and outcomes. Psychological Medicine. 2010;40(12):2049-57.

6. HMGovernment. Data protection and sharing - guidance for emergency planners and responders. London: Cabinet Office; 2007.

7. Close RM, Maguire H, Etherington G, Brewin CR, Fong K, Saliba V, et al. Preparedness for a major incident: Creation of an epidemiology protocol for a health protection register in England. Environment International. 2014;72:75-82.

8. Ipsos MORI. Public attitudes to the use and sharing of their data. Research for the Royal Statistical Society by Ipsos MORI. London: Ipsos MORI; 2014.

9. Barrett G, Cassell JA, Peacock JL, Coleman MP, National Cancer R. National survey of British public's views on use of identifiable medical data by the National Cancer Registry. Bmj. 2006;332(7549):1068-72.

10. Kaufman DJ, Murphy-Bollinger J, Scott J, Hudson KL. Public opinion about the importance of privacy in biobank research. American journal of human genetics. 2009;85(5):643-54.

11. National Readership Survey. National Readership Survey 2015. 2015.

12. Office for National Statistics. Internet users: 2015. Available from: https://www.ons.gov.uk/businessindustryandtrade/itandinternetindustry/bulletins/internetusers/2015 2015.

For peer review only - http://bmjopen.bmj.com/site/about/guidelines.xhtml

Table 1. Demographic characteristics of survey respondents

		Frequency (%)		
**Variable**	**Categories**	**Pre-incidents**	**Post-incidents**	**Total**
Sex	Male	490 (49.0)	496 (49.6)	986 (49.3)
	Female	510 (51.0)	504 (50.4)	1014 (50.7)
Age	18 to 24	119 (11.9)	144 (14.4)	263 (13.2)
	25 to 34	225 (22.5)	222 (22.2)	447 (22.4)
	35 to 44	213 (21.3)	207 (20.7)	420 (21.0)
	45 to 54	233 (23.3)	226 (22.6)	459 (23.0)
	55 to 65	210 (21.0)	201 (20.1)	411 (20.6)
Region	North East	46 (4.6)	46 (4.6)	92 (4.6)
	North West	137 (13.7)	135 (13.5)	272 (13.6)
	Yorkshire and Humberside	98 (9.8)	101 (10.1)	199 (10.0)
	West Midlands	97 (9.7)	101 (10.1)	198 (9.9)
	East Midlands	89 (8.9)	84 (8.4)	173 (8.7)
	East of England	90 (9.0)	99 (9.9)	189 (9.5)
	South West	95 (9.5)	90 (9.0)	185 (9.3)
	South East	167 (16.7)	166 (16.6)	333 (16.7)
	London	181 (18.1)	178 (17.8)	359 (18.0)
Employment status	Working full time	501 (50.1)	509 (50.9)	1010 (50.5)
	Working part time	151 (15.1)	140 (14.0)	291 (14.6)
	Self-employed	70 (7.0)	78 (7.8)	148 (7.4)
	Unemployed, looking for a job	49 (4.9)	52 (5.2)	101 (5.1)
	Unemployed not looking for a job	105 (10.5)	103 (10.3)	208 (10.4)
	Retired	78 (7.8)	56 (5.6)	134 (6.7)
	Pupil / student / in full-time education	46 (4.6)	62 (6.2)	108 (5.4)
Highest level of education achieved	Left school without qualifications	33 (3.3)	27 (2.7)	60 (3.0)
	Secondary education	424 (42.4)	413 (41.3)	837 (41.9)
	Higher education	538 (53.8)	555 (55.5)	1093 (54.7)
	Prefer not to say	5 (0.5)	5 (0.5)	10 (0.5)

For peer review only - http://bmjopen.bmj.com/site/about/guidelines.xhtml

Table 2. Ability of police sharing personal data with other agencies, without asking first

Agency requesting personal information and reason for the request		Frequency (%) believing if police are able to share for this reason						
		Shooting	Radiation	χ^2	Pre-incidents	Post-incidents	χ^2	Total
Your GP, so they can check how you are doing	Able	497 (49.7)	581 (58.1)		541 (54.1)	537 (53.7)		1078 (53.9)
	Not sure	212 (21.2)	203 (20.3)	17.835***	206 (20.6)	209 (20.9)	0.039	415 (20.8)
	Not able	291 (29.1)	216 (21.6)		253 (25.3)	254 (25.4)		507 (25.4)
Your travel insurance company, so they can offer you practical and financial support	Able	332 (33.2)	323 (32.3)		320 (32.0)	335 (33.5)		655 (32.8)
	Not sure	254 (25.4)	263 (26.3)	0.280	271 (27.1)	246 (24.6)	1.673	517 (25.9)
	Not able	414 (41.4)	414 (41.4)		409 (40.9)	419 (41.9)		828 (41.4)
A team of medical researchers from a university, so they can invite you to take part in a study to improve the way future incidents are dealt with	Able	161 (16.1)	201 (20.1)		186 (18.6)	176 (17.6)		362 (18.1)
	Not sure	226 (22.6)	232 (23.3)	6.292*	221 (22.1)	237 (23.7)	0.866	458 (22.9)
	Not able	613 (61.3)	567 (56.7)		593 (59.3)	587 (58.7)		1180 (59.0)
A journalist, so they can write a news article about the incident	Able	79 (7.9)	86 (8.6)		79 (7.9)	86 (8.6)		165 (8.3)
	Not sure	132 (13.2)	142 (14.2)	0.847	133 (13.3)	141 (14.1)	0.675	274 (13.7)
	Not able	789 (78.9)	772 (77.2)		788 (78.8)	773 (77.3)		1561 (78.0)
A charity such as the British Red Cross, so they can offer you support	Able	256 (25.6)	255 (25.5)		243 (24.3)	268 (26.8)		511 (25.6)
	Not sure	281 (28.1)	272 (27.2)	0.255	281 (28.1)	272 (27.2)	1.643	553 (27.7)
	Not able	463 (46.3)	473 (47.3)		476 (47.6)	460 (46.0)		936 (46.8)
A health-related Government organisation, so they can send you a questionnaire to find out if you might benefit from extra care or support	Able	409 (40.9)	509 (50.9)		462 (46.2)	456 (45.6)		918 (45.9)
	Not sure	283 (28.3)	249 (24.9)	20.986***	266 (26.6)	266 (26.6)	0.105	532 (26.6)
	Not able	308 (30.8)	242 (24.2)		272 (27.2)	278 (27.8)		550 (27.5)
A specialist NHS team, to provide you with information about ways to get support for any physical or mental health issues	Able	563 (56.3)	653 (65.3)		608 (60.8)	608 (60.8)		1216 (60.8)
	Not sure	218 (21.8)	195 (19.5)	20.042***	209 (20.9)	204 (20.4)	0.128	413 (20.7)
	Not able	219 (21.9)	152 (15.2)		183 (18.3)	188 (18.8)		371 (18.6)
A law firm, so they can offer to represent you in a ‘no-win, no-fee’ claim for compensation	Able	128 (12.8)	127 (12.7)		127 (12.7)	128 (12.8)		255 (12.8)
	Not sure	164 (16.4)	197 (19.7)	3.760	182 (18.2)	179 (17.9)	0.032	361 (18.1)
	Not able	708 (70.8)	676 (67.6)		691 (69.1)	693 (69.3)		1384 (69.2)

Note: Able = those that thought the police we probably or definitely able, Not able = those that thought the police were probably or definitely not able, *p<.05, ***p<.001

For peer review only - http://bmjopen.bmj.com/site/about/guidelines.xhtml

Table 3. Acceptability of police sharing personal data with other agencies, without asking first

Agency requesting personal information and reason for the request		Frequency (%) believing if it is acceptable for the police to share for this reason						
		Shooting	Radiation	χ^2	Pre-incidents	Post-incidents	χ^2	Total
Your GP, so they can check how you are doing	Acceptable	674 (67.4)	728 (72.8)		691 (69.1)	711 (71.1)		1402 (70.1)
	Not sure	131 (13.1)	120 (12.0)	7.890*	121 (12.1)	130 (13.0)	3.032	251 (12.6)
	Not acceptable	195 (19.5)	152 (15.2)		188 (18.8)	159 (15.9)		347 (17.4)
Your travel insurance company, so they can offer you practical and financial support	Acceptable	398 (39.8)	385 (38.5)		387 (38.7)	396 (39.6)		783 (39.1)
	Not sure	214 (21.4)	222 (22.2)	0.395	232 (23.2)	204 (20.4)	2.364	436 (21.8)
	Not acceptable	388 (38.8)	393 (39.3)		381 (38.1)	400 (40.0)		781 (39.1)
A team of medical researchers from a university, so they can invite you to take part in a study to improve the way future incidents are dealt with	Acceptable	216 (21.6)	276 (27.6)		266 (26.6)	226 (22.6)		492 (24.6)
	Not sure	221 (22.1)	238 (23.8)	13.599**	214 (21.4)	245 (24.5)	5.423	459 (22.9)
	Not acceptable	563 (56.3)	486 (48.6)		520 (52.0)	529 (53.9)		1049 (52.5)
A journalist, so they can write a news article about the incident	Acceptable	59 (5.9)	66 (6.6)		59 (5.9)	66 (6.6)		125 (6.3)
	Not sure	85 (8.5)	100 (10.0)	1.895	84 (8.4)	101 (10.1)	2.295	185 (9.3)
	Not acceptable	856 (85.6)	834 (83.4)		857 (85.7)	833 (833)		1690 (84.5)
A charity such as the British Red Cross, so they can offer you support	Acceptable	349 (34.9)	366 (36.6)		345 (34.5)	370 (37.0)		715 (35.8)
	Not sure	260 (26.0)	245 (24.5)	0.855	254 (25.4)	251 (25.1)	1.512	505 (25.3)
	Not acceptable	391 (39.1)	389 (38.9)		401 (40.1)	379 (37.9)		780 (39.0)
A health-related Government organisation, so they can send you a questionnaire to find out if you might benefit from extra care or support	Acceptable	478 (47.8)	535 (53.5)		493 (49.3)	520 (52.0)		1013 (50.6)
	Not sure	229 (22.9)	208 (20.8)	6.573*	225 (22.5)	212 (21.2)	1.463	437 (21.9)
	Not acceptable	293 (29.3)	257 (25.7)		282 (28.2)	268 (26.8)		550 (27.5)
A specialist NHS team, to provide you with information about ways to get support for any physical or mental health issues	Acceptable	696 (69.6)	742 (74.2)		711 (71.1)	727 (72.7)		1438 (71.9)
	Not sure	138 (13.8)	126 (12.6)	5.896	139 (13.9)	125 (12.5)	0.934	264 (13.2)
	Not acceptable	166 (16.6)	132 (13.2)		150 (15.0)	148 (14.8)		298 (14.9)
A law firm, so they can offer to represent you in a 'no-win, no-fee' claim for compensation	Acceptable	107 (10.7)	113 (11.3)	0.475	115 (11.5)	105 (10.5)		220 (11.0)
	Not sure	137 (13.7)	128 (12.8)		127 (12.7)	138 (13.8)	0.912	265 (13.3)
	Not acceptable	756 (75.6)	759 (75.9)		758 (75.8)	757 (75.7)		1515 (75.8)

Note: Acceptable = those that thought it was probably or definitely acceptable, Not acceptable = those that thought it was probably or definitely not acceptable, *p<.05, **p<.01.

For peer review only - http://bmjopen.bmj.com/site/about/guidelines.xhtml

Table 4. Attitudes towards proposed confidential database

If I was caught up in a terrorist incident		Frequency (%) of agreement			
		Pre-incidents	Post-incidents	χ^2	Total
I would want the police to give my contact details to the government organisation so that they can contact me about this service	Agree	663 (66.3)	669 (66.9)		1332 (66.6)
	Neither	177 (17.7)	175 (17.5)	0.089	352 (17.6)
	Disagree	160 (16.0)	156 (15.6)		316 (15.8)
I would want my name to be included on this database	Agree	531 (53.1)	501 (50.1)		1032 (51.6)
	Neither	242 (24.2)	265 (26.5)	2.022	507 (25.4)
	Disagree	227 (22.7)	234 (23.4)		461 (23.1)
I would be unhappy if my name was included on this database	Agree	270 (27.0)	287 (28.7)		557 (27.9)
	Neither	305 (30.5)	280 (28.0)	1.662	585 (29.3)
	Disagree	425 (42.5)	433 (43.3)		858 (42.9)

Note: Agree = those that selected tend to or strongly agree, Disagree = those that selected tend to or strongly disagree. This data was not split by scenario type, as it regarded a different scenario to that originally posed to participants.

For peer review only

For peer review only - http://bmjopen.bmj.com/site/about/guidelines.xhtml

Table 5. Attitudes towards contact details being shared with another agency by the police

Imagine the scenario we described occurred and the police decided to share your name and contact information with a health-related government organisation		Frequency (%) of agreement						
		Shooting	Radiation	χ^2	Pre-incidents	Post-incidents	χ^2	Total
I would be concerned that my details would be made public by accident	Agree	650 (65.0)	602 (60.2)		624 (62.4)	628 (62.8)		1252 (62.6)
	Neither	201 (20.1)	223 (22.3)	5.068	217 (21.7)	207 (20.7)	0.360	424 (21.2)
	Disagree	149 (14.9)	175 (17.5)		159 (15.9)	165 (16.5)		324 (16.2)
I would trust the health organisation to keep my details secure	Agree	515 (51.5)	572 (57.2)		546 (54.6)	541 (54.1)		1087 (54.4)
	Neither	266 (26.6)	245 (24.5)	7.076*	256 (25.6)	255 (25.5)	0.115	511 (25.6)
	Disagree	219 (21.9)	183 (18.3)		198 (19.8)	204 (20.4)		402 (20.1)
It would be an invasion of my privacy	Agree	446 (44.6)	386 (38.6)		410 (41.0)	422 (42.2)		832 (41.6)
	Neither	306 (30.6)	288 (28.8)	15.472***	293 (29.3)	301 (30.1)	0.978	594 (29.7)
	Disagree	248 (24.8)	326 (32.6)		297 (29.7)	277 (27.7)		574 (28.7)
I would be concerned about how my information might be used in the future	Agree	691 (69.1)	665 (66.5)		671 (67.1)	685 (68.5)		1356 (67.8)
	Neither	177 (17.7)	203 (20.3)	2.277	192 (19.2)	188 (18.8)	0.565	380 (19.0)
	Disagree	132 (13.2)	132 (13.2)		137 (13.7)	127 (12.7)		264 (13.2)
I would be concerned that my employer would be told information about me	Agree	348 (34.8)	336 (33.6)		343 (34.3)	341 (34.1)		684 (34.2)
	Neither	281 (28.1)	298 (29.8)	0.744	284 (28.4)	295 (29.5)	0.325	579 (28.9)
	Disagree	371 (37.1)	366 (366)		373 (37.3)	364 (36.4)		737 (36.9)
I would be concerned that my details might be shared by the health organisation with other groups without my permission	Agree	657 (65.7)	613 (61.3)		627 (62.7)	643 (64.3)		1270 (63.5)
	Neither	201 (20.1)	245 (24.5)	5.865	227 (22.7)	219 (21.9)	0.570	446 (22.3)
	Disagree	142 (14.2)	142 (14.2)		146 (14.6)	128 (13.8)		284 (14.2)
I would want to be kept informed about how my information was being used	Agree	849 (84.9)	846 (84.6)		857 (85.7)	838 (83.8)		1695 (84.8)
	Neither	114 (11.4)	128 (12.8)	2.736	109 (10.9)	133 (13.3)	2.990	242 (12.1)
	Disagree	37 (3.7)	26 (2.6)		34 (3.4)	29 (2.9)		63 (3.2)
It wouldn't bother me at all	Agree	262 (26.2)	294 (29.4)		266 (26.6)	290 (29.0)		556 (27.8)
	Neither	308 (30.8)	330 (33.0)	6.218*	332 (33.2)	306 (30.6)	2.100	638 (31.9)
	Disagree	430 (43.0)	376 (37.6)		402 (40.2)	404 (40.4)		806 (40.3)
I would be reassured that a health organisation was looking out for me	Agree	532 (53.2)	575 (57.5)		552 (55.2)	555 (55.5)		1107 (55.4)
	Neither	305 (30.5)	302 (30.2)	7.280*	319 (31.9)	288 (28.8)	4.333	607 (60.7)
	Disagree	163 (16.3)	123 (12.3)		129 (12.9)	157 (15.7)		286 (14.3)
I would be concerned that my GP might be told	Agree	146 (14.6)	131 (13.1)		131 (13.1)	146 (14.6)		277 (13.8)
	Neither	296 (29.6)	277 (27.7)	2.448	292 (29.2)	281 (28.1)	1.037	573 (28.7)
	Disagree	558 (55.8)	592 (59.2)		577 (57.7)	573 (57.3)		1150 (57.5)

Note: Agree = those that selected tend to or strongly agree, Disagree = those that selected tend to or strongly disagree, *p<.05, ***p<.001

For peer review only - http://bmjopen.bmj.com/site/about/guidelines.xhtml

Supplementary material 1: KCL Disaster Data Protection Topline Results for Wave 1Supplementary material 1 - KCL Disaster Data Protection Topline Results for Wave 1

KCL Disaster Data Protection Survey
Topline results

- Results are based on interviews with 1000 members of the public aged 18-65 across England. Interviews were conducted between 9 March 2017 and 15 March 2017.
- All interviews were conducted via Ipsos MORI's online panel.
- The survey data is unweighted but based on quotas on age, gender, region and working status to reflect the profile of adults aged 18-65 in England, with up to 10% flexibility. Weights are available in the SPSS file for the whole sample, Sample A and Sample B.
- Results are based on all respondents (1000) unless otherwise stated. Bases specified are unweighted.
- Where percentages do not sum to 100, this may be due to respondents being able to select multiple responses, computer rounding or the exclusion of 'don't know'/ not stated.
- An asterisk (*) represents a value of less than half or one percent, but greater than zero.
- The response rate for this survey was 10% (1000 responses from 10004 people that the link was emailed to).
- 25 participants ended the survey without completing it (and were not included in the data); 26 people were excluded from the data due to being identified as 'speeding' or straightlining'; i.e. completing the survey too quickly to have given genuine, considered answers, or providing identical answers to five or more consecutive questions where this was possible.

Q1. Please select your date of birth (recoded into age categories):

	%
18-24	12
25-34	23
35-44	21
45-54	23
55-65	21

Q2. Please state the gender you identify yourself with:

	%
Male	49
Female	51

Q3. In which of the following regions do you live?

	%
North East	5
North West	14
Yorkshire and Humberside	10
West Midlands	10
East Midlands	9
East of England	9
South West	10
South East	17
London	18

For peer review only - http://bmjopen.bmj.com/site/about/guidelines.xhtml

Supplementary material 1: KCL Disaster Data Protection Topline Results for Wave 1Supplementary material 1 - KCL Disaster Data Protection Topline Results for Wave 1

Q4. Which of the following best describes your employment status?

	%
Working – full time (30 or more hours a week)	50
Working – part-time (less than 30 hours a week)	15
Self-employed	7
Unemployed – looking for a job	5
Unemployed – not looking for a job/long-term sick or disabled/Housewife/husband/Full-time carer	11
Retired	8
Pupil/Student/In full-time education	5
**NET: Working**	72
**NET: Not working**	28

We are interested in people's views about sharing information following a major incident such as a terrorist attack. The researchers have no reason to think that an attack is going to happen. This research is one of a number of studies that academics at King's College London are conducting on how people might react to a range of different issues.

SAMPLE A:
Imagine that you are on holiday in another country and witness a terrorist shooting. Some people are badly injured but you are not harmed and neither is anyone you know well. The British government arranges for you to be flown home, along with other British nationals who were in the area. When you arrive back in Britain, a police officer at the airport records your name, address, phone number and email address. The following questions ask about things that might happen next.

SAMPLE B:
Imagine that you are on holiday in another country and a place you visit is discovered by police to be contaminated by radioactive material. The police believe this is linked to a terrorist group. Some people are badly injured but you are not harmed and neither is anyone you know well. The British government arranges for you to be flown home, along with other British nationals who were in the area. When you arrive back in Britain, a police officer at the airport records your name, address, phone number and email address. The following questions ask about things that might happen next.

Q5. (TOTAL) Following an incident like this, various groups or individuals might ask the police to tell them who was caught up in the incident. Please say whether you think that the police are or are not currently able to share your name and contact details with these groups or people following an incident like this, without checking with you first.

1. **Your GP, so they can check how you are doing**

	% (TOTAL – Base = 1000)	% (SAMPLE A – Base = 500)	% (SAMPLE B – Base = 500)
Definitely able to share	18	14	22
Probably able to share	36	37	35
Not sure	21	21	20
Probably not able to share	15	18	13
Definitely not able to share	10	10	10

2. **Your travel insurance company, so they can offer you practical and financial support**

	% (TOTAL – Base = 1000)	% (SAMPLE A – Base = 500)	% (SAMPLE B – Base = 500)
Definitely able to share	8	8	9
Probably able to share	24	25	23
Not sure	27	27	28
Probably not able to share	24	26	22

For peer review only - http://bmjopen.bmj.com/site/about/guidelines.xhtml

Supplementary material 1: KCL Disaster Data Protection Topline Results for Wave 1Supplementary material 1 - KCL Disaster Data Protection Topline Results for Wave 1

Definitely not able to share	17	16	19

3. A team of medical researchers from a university, so they can invite you to take part in a study to improve the way future incidents are dealt with

	% (TOTAL – Base = 1000)	% (SAMPLE A – Base = 500)	% (SAMPLE B – Base = 500)
Definitely able to share	5	4	6
Probably able to share	14	14	13
Not sure	22	22	23
Probably not able to share	31	31	30
Definitely not able to share	28	29	28

4. A journalist, so they can write a news article about the incident

	% (TOTAL – Base = 1000)	% (SAMPLE A – Base = 500)	% (SAMPLE B – Base = 500)
Definitely able to share	3	3	2
Probably able to share	5	4	7
Not sure	13	13	13
Probably not able to share	20	22	19
Definitely not able to share	59	58	59

5. A charity, such as the British Red Cross, so they can offer you support

	% (TOTAL – Base = 1000)	% (SAMPLE A – Base = 500)	% (SAMPLE B – Base = 500)
Definitely able to share	6	5	7
Probably able to share	18	20	16
Not sure	28	29	28
Probably not able to share	29	28	30
Definitely not able to share	19	18	20

6. A health-related Government organisation, so they can send you a questionnaire to find out if you might benefit from extra care or support.

	% (TOTAL – Base = 1000)	% (SAMPLE A – Base = 500)	% (SAMPLE B – Base = 500)
Definitely able to share	10	8	13
Probably able to share	36	33	39
Not sure	27	29	25
Probably not able to share	16	18	15
Definitely not able to share	11	13	9

7. A specialist NHS team, to provide you with information about ways to get support for any physical or mental health issues.

	% (TOTAL – Base = 1000)	% (SAMPLE A – Base = 500)	% (SAMPLE B – Base = 500)
Definitely able to share	18	14	22
Probably able to share	43	41	45
Not sure	21	25	17
Probably not able to share	11	14	9
Definitely not able to share	7	7	7

For peer review only - http://bmjopen.bmj.com/site/about/guidelines.xhtml

Supplementary material 1: KCL Disaster Data Protection Topline Results for Wave 1Supplementary material 1 - KCL Disaster Data Protection Topline Results for Wave 1

8. A law firm, so they can offer to represent you in a 'no-win, no-fee' claim for compensation

	% (TOTAL – Base = 1000)	% (SAMPLE A – Base = 500)	% (SAMPLE B – Base = 500)
Definitely able to share	4	3	4
Probably able to share	9	9	9
Not sure	18	17	20
Probably not able to share	25	23	27
Definitely not able to share	44	48	41

Q6. Following an incident like this, various groups or individuals might ask the police to tell them who was caught up in the incident. For each of the following groups, please say whether you think that is acceptable or not acceptable for the police to share your name and contact details with these groups or people following an incident like this, without checking with you first;

1. Your GP, so they can check how you are doing

	% (TOTAL – Base = 1000)	% (SAMPLE A – Base = 500)	% (SAMPLE B – Base = 500)
Definitely acceptable to share	30	26	33
Probably acceptable to share	39	38	40
Not sure	12	14	10
Probably not acceptable to share	10	11	9
Definitely not acceptable to share	9	10	8

2. Your travel insurance company, so they can offer you practical and financial support

	% (TOTAL – Base = 1000)	% (SAMPLE A – Base = 500)	% (SAMPLE B – Base = 500)
Definitely acceptable to share	11	9	13
Probably acceptable to share	27	30	25
Not sure	23	23	24
Probably not acceptable to share	16	16	16
Definitely not acceptable to share	22	22	22

3. A team of medical researchers from a university, so they can invite you to take part in a study to improve the way future incidents are dealt with

	% (TOTAL – Base = 1000)	% (SAMPLE A – Base = 500)	% (SAMPLE B – Base = 500)
Definitely acceptable to share	6	5	8
Probably acceptable to share	20	19	22
Not sure	21	22	21
Probably not acceptable to share	23	23	23
Definitely not acceptable to share	29	31	27

4. A journalist, so they can write a news article about the incident

	% (TOTAL – Base = 1000)	% (SAMPLE A – Base = 500)	% (SAMPLE B – Base = 500)
Definitely acceptable to share	2	1	2
Probably acceptable to share	4	4	4
Not sure	8	10	7
Probably not acceptable to share	14	13	14
Definitely not acceptable to share	72	72	73

For peer review only - http://bmjopen.bmj.com/site/about/guidelines.xhtml

Supplementary material 1: KCL Disaster Data Protection Topline Results for Wave 1Supplementary material 1 - KCL Disaster Data Protection Topline Results for Wave 1

5. A charity, such as the British Red Cross, so they can offer you support

	% (TOTAL – Base = 1000)	% (SAMPLE A – Base = 500)	% (SAMPLE B – Base = 500)
Definitely acceptable to share	8	7	8
Probably acceptable to share	27	27	27
Not sure	25	27	24
Probably not acceptable to share	20	19	20
Definitely not acceptable to share	20	20	20

6. A health-related Government organisation, so they can send you a questionnaire to find out if you might benefit from extra care or support.

	% (TOTAL – Base = 1000)	% (SAMPLE A – Base = 500)	% (SAMPLE B – Base = 500)
Definitely acceptable to share	13	10	17
Probably acceptable to share	36	35	37
Not sure	23	25	20
Probably not acceptable to share	15	16	15
Definitely not acceptable to share	13	14	12

7. A specialist NHS team, to provide you with information about ways to get support for any physical or mental health issues.

	% (TOTAL – Base = 1000)	% (SAMPLE A – Base = 500)	% (SAMPLE B – Base = 500)
Definitely acceptable to share	27	23	31
Probably acceptable to share	44	43	45
Not sure	14	16	12
Probably not acceptable to share	8	9	7
Definitely not acceptable to share	7	8	6

8. A law firm, so they can offer to represent you in a 'no-win, no-fee' claim for compensation

	% (TOTAL – Base = 1000)	% (SAMPLE A – Base = 500)	% (SAMPLE B – Base = 500)
Definitely acceptable to share	3	3	4
Probably acceptable to share	8	8	9
Not sure	13	14	11
Probably not acceptable to share	16	14	18
Definitely not acceptable to share	60	61	59

Following a terrorist incident, people sometimes develop mental health problems as a result of their experiences. To provide help, the Government sometimes sets up a specialist NHS service to offer support to those who need it. A government organisation would be asked to help with this by putting together a confidential database of people who were present and then writing to them with information about the NHS service and a short mental health questionnaire.

Q7. To what extent do you agree or disagree with the following statement. If I was caught up in a terrorist incident, I would want the police to give my contact details to the government organisation so that they can contact me about this service.

	%
Strongly agree	21
Tend to agree	45
Neither agree nor disagree	18
Tend to disagree	12
Strongly disagree	4

For peer review only - http://bmjopen.bmj.com/site/about/guidelines.xhtml

Supplementary material 1: KCL Disaster Data Protection Topline Results for Wave 1Supplementary material 1 - KCL Disaster Data Protection Topline Results for Wave 1

Q8. And why do you say that? OPEN-ENDED RESPONSES

Q9. To what extent do you agree or disagree with the following statement: If I was caught up in a terrorist incident, I would want my name to be included on this database.

	%
Strongly agree	14
Tend to agree	39
Neither agree nor disagree	24
Tend to disagree	16
Strongly disagree	7

Q10. To what extent do you agree or disagree with the following statement: If I was caught up in a terrorist incident, I would be unhappy if my name was included on this database.

	%
Strongly agree	9
Tend to agree	18
Neither agree nor disagree	31
Tend to disagree	32
Strongly disagree	10

Q11A. Imagine the scenario we described occurred and the police decided to share your name and contact information with a health-related government organisation. Please say how much you agree or disagree with the following statements:

1. **I would be concerned that my details would be made public by accident**

	% (TOTAL – Base = 1000)	% (SAMPLE A – Base = 500)	% (SAMPLE B – Base = 500)
Strongly agree	26	28	24
Tend to agree	37	38	35
Neither agree nor disagree	22	20	23
Tend to disagree	14	13	14
Strongly disagree	2	2	3

2. **I would trust the health organisation to keep my details secure**

	% (TOTAL – Base = 1000)	% (SAMPLE A – Base = 500)	% (SAMPLE B – Base = 500)
Strongly agree	17	16	19
Tend to agree	38	36	39
Neither agree nor disagree	26	27	25
Tend to disagree	15	18	13
Strongly disagree	5	4	5

3. **It would be an invasion of my privacy**

	% (TOTAL – Base = 1000)	% (SAMPLE A – Base = 500)	% (SAMPLE B – Base = 500)
Strongly agree	16	17	15
Tend to agree	25	26	23
Neither agree nor disagree	29	32	27
Tend to disagree	24	21	28
Strongly disagree	5	4	6

For peer review only - http://bmjopen.bmj.com/site/about/guidelines.xhtml

Supplementary material 1: KCL Disaster Data Protection Topline Results for Wave 1Supplementary material 1 - KCL Disaster Data Protection Topline Results for Wave 1

4. I would be concerned about how my information might be used in the future

	% (TOTAL – Base = 1000)	% (SAMPLE A – Base = 500)	% (SAMPLE B – Base = 500)
Strongly agree	27	30	24
Tend to agree	41	41	40
Neither agree nor disagree	19	16	22
Tend to disagree	12	11	12
Strongly disagree	2	2	2

5. I would be concerned that my employer would be told information about me

	% (TOTAL – Base = 1000)	% (SAMPLE A – Base = 500)	% (SAMPLE B – Base = 500)
Strongly agree	12	12	11
Tend to agree	23	25	21
Neither agree nor disagree	28	28	29
Tend to disagree	27	25	29
Strongly disagree	11	11	11

Q11B. Imagine the scenario we described occurred and the police decided to share your name and contact information with a health-related government organisation. Please say how much you agree or disagree with the following statements:

1. I would be concerned that my details might be shared by the health organisation with other groups without my permission

	% (TOTAL – Base = 1000)	% (SAMPLE A – Base = 500)	% (SAMPLE B – Base = 500)
Strongly agree	25	27	22
Tend to agree	38	36	41
Neither agree nor disagree	23	22	23
Tend to disagree	11	11	12
Strongly disagree	3	4	3

2. I would want to be kept informed about how my information was being used

	% (TOTAL – Base = 1000)	% (SAMPLE A – Base = 500)	% (SAMPLE B – Base = 500)
Strongly agree	52	52	51
Tend to agree	34	32	35
Neither agree nor disagree	11	11	11
Tend to disagree	3	3	2
Strongly disagree	1	1	*

3. It wouldn't bother me at all

	% (TOTAL – Base = 1000)	% (SAMPLE A – Base = 500)	% (SAMPLE B – Base = 500)
Strongly agree	6	5	7
Tend to agree	20	19	21
Neither agree nor disagree	33	32	35
Tend to disagree	26	28	24
Strongly disagree	15	16	13

For peer review only - http://bmjopen.bmj.com/site/about/guidelines.xhtml

Supplementary material 1: KCL Disaster Data Protection Topline Results for Wave 1Supplementary material 1 - KCL Disaster Data Protection Topline Results for Wave 1

4. I would be reassured that a health organisation was looking out for me

	% (TOTAL – Base = 1000)	% (SAMPLE A – Base = 500)	% (SAMPLE B – Base = 500)
Strongly agree	14	11	17
Tend to agree	42	43	40
Neither agree nor disagree	32	32	31
Tend to disagree	9	10	8
Strongly disagree	4	4	4

5. I would be concerned that my GP might be told

	% (TOTAL – Base = 1000)	% (SAMPLE A – Base = 500)	% (SAMPLE B – Base = 500)
Strongly agree	4	4	4
Tend to agree	9	11	7
Neither agree nor disagree	29	30	28
Tend to disagree	40	38	41
Strongly disagree	18	17	19

Q12. Please state the highest level of education you have achieved:

	%
Left school without qualifications	3
Secondary education (O-level/GCSE/A-level)	42
Higher education (BSc, BA/higher qualification)	54
Prefer not to say	1

For peer review only

Supplementary material 2 - KCL Disaster Data Protection Topline Results for Wave 2

KCL Disaster Data Protection Survey – Wave 2
Topline results

- Results are based on interviews with 1000 members of the public aged 18-65 across England. Interviews were conducted between 3 and 10 July 2017.
- All interviews were conducted via Ipsos MORI's online panel.
- The survey data is unweighted but based on quotas on age, gender, region and working status (with age and gender interlocked) to reflect the profile of adults aged 18-65 in England, with up to 10% flexibility on region and working status. Weights are available in the SPSS file for the whole sample, Sample A and Sample B, for both the first and second wave and for both waves combined.
- Results are based on all respondents (1000) unless otherwise stated. Bases specified are unweighted.
- Where percentages do not sum to 100, this may be due to respondents being able to select multiple responses, computer rounding or the exclusion of 'don't know'/ not stated.
- An asterisk (*) represents a value of less than half of one percent, but greater than zero.
- 14,735 people were invited to take part in the survey.
- 30 participants ended the survey without completing it (and were not included in the data); 19 people were excluded from the data due to being identified as 'speeding' or straightlining'; i.e. completing the survey too quickly to have given genuine, considered answers, or providing identical answers to five or more consecutive questions where this was possible.
- This work has been carried out in accordance with the requirements of the international quality standard for Market Research, ISO 20252:2012, and with the standard Ipsos MORI Terms and Conditions which can be found at http://www.ipsos-mori.com/terms. © Ipsos MORI 2017

Q1. Please select your date of birth (recoded into age categories):

	%
18-24	14.4
25-34	22.2
35-44	20.7
45-54	22.6
55-65	20.1

Q2. Please state the gender you identify yourself with:

	%
Male	49.6
Female	50.4

Q3. In which of the following regions do you live?

	%
North East	4.6
North West	13.5
Yorkshire and Humberside	10.1
West Midlands	10.1
East Midlands	8.4
East of England	9.9
South West	9.0
South East	16.6

For peer review only - http://bmjopen.bmj.com/site/about/guidelines.xhtml

Supplementary material 2 - KCL Disaster Data Protection Topline Results for Wave 2

London	17.8

Q4. Which of the following best describes your employment status?

	%
Working – full time (30 or more hours a week)	50.9
Working – part-time (less than 30 hours a week)	14.0
Self-employed	7.8
Unemployed – looking for a job	5.2
Unemployed – not looking for a job/long-term sick or disabled/Housewife/husband/Full-time carer	10.3
Retired	5.6
Pupil/Student/In full-time education	6.2
**NET: Working**	72.7
**NET: Not working**	27.3

We are interested in people's views about sharing information following a major incident such as a terrorist attack. The researchers have no reason to think that an attack is going to happen. This research is one of a number of studies that academics at King's College London are conducting on how people might react to a range of different issues.

SAMPLE A:
Imagine that you are on holiday in another country and witness a terrorist shooting. Some people are badly injured but you are not harmed and neither is anyone you know well. The British government arranges for you to be flown home, along with other British nationals who were in the area. When you arrive back in Britain, a police officer at the airport records your name, address, phone number and email address. The following questions ask about things that might happen next.

SAMPLE B:
Imagine that you are on holiday in another country and a place you visit is discovered by police to be contaminated by radioactive material. The police believe this is linked to a terrorist group. Some people are badly injured but you are not harmed and neither is anyone you know well. The British government arranges for you to be flown home, along with other British nationals who were in the area. When you arrive back in Britain, a police officer at the airport records your name, address, phone number and email address. The following questions ask about things that might happen next.

Q5. Following an incident like this, various groups or individuals might ask the police to tell them who was caught up in the incident. Please say whether you think that the police are or are not currently able to share your name and contact details with these groups or people following an incident like this, without checking with you first.

1. **Your GP, so they can check how you are doing**

	% (TOTAL – Base = 1000)	% (SAMPLE A – Base = 500)	% (SAMPLE B – Base = 500)
Definitely able to share	19.6	19.8	19.4
Probably able to share	34.1	28.8	39.4
Not sure	20.9	21.2	20.6
Probably not able to share	15.2	18.8	11.6
Definitely not able to share	10.2	11.4	9.0

2. **Your travel insurance company, so they can offer you practical and financial support**

	% (TOTAL – Base = 1000)	% (SAMPLE A – Base = 500)	% (SAMPLE B – Base = 500)
Definitely able to share	10.6	11.8	9.4
Probably able to share	22.9	22.4	23.4
Not sure	24.6	24.2	25.0
Probably not able to share	23.2	22.8	23.6

For peer review only - http://bmjopen.bmj.com/site/about/guidelines.xhtml

Supplementary material 2 - KCL Disaster Data Protection Topline Results for Wave 2

Definitely not able to share	18.7	18.8	18.6

3. A team of medical researchers from a university, so they can invite you to take part in a study to improve the way future incidents are dealt with

	% (TOTAL – Base = 1000)	% (SAMPLE A – Base = 500)	% (SAMPLE B – Base = 500)
Definitely able to share	4.4	3.8	5.0
Probably able to share	13.2	10.6	15.8
Not sure	23.7	23.6	23.8
Probably not able to share	30.1	31.4	28.8
Definitely not able to share	28.6	30.6	26.6

4. A journalist, so they can write a news article about the incident

	% (TOTAL – Base = 1000)	% (SAMPLE A – Base = 500)	% (SAMPLE B – Base = 500)
Definitely able to share	2.3	2.4	2.2
Probably able to share	6.3	6.2	6.4
Not sure	14.1	13.2	15.0
Probably not able to share	20.7	19.0	22.4
Definitely not able to share	56.6	59.2	54.0

5. A charity, such as the British Red Cross, so they can offer you support

	% (TOTAL – Base = 1000)	% (SAMPLE A – Base = 500)	% (SAMPLE B – Base = 500)
Definitely able to share	5.7	5.6	5.8
Probably able to share	21.1	20.0	22.2
Not sure	27.2	27.6	26.8
Probably not able to share	26.5	25.6	27.4
Definitely not able to share	19.5	21.2	17.8

6. A health-related Government organisation, so they can send you a questionnaire to find out if you might benefit from extra care or support.

	% (TOTAL – Base = 1000)	% (SAMPLE A – Base = 500)	% (SAMPLE B – Base = 500)
Definitely able to share	10.7	11.0	10.4
Probably able to share	34.9	29.8	40.0
Not sure	26.6	28.0	25.2
Probably not able to share	17.1	19.4	14.8
Definitely not able to share	10.7	11.8	9.6

7. A specialist NHS team, to provide you with information about ways to get support for any physical or mental health issues.

	% (TOTAL – Base = 1000)	% (SAMPLE A – Base = 500)	% (SAMPLE B – Base = 500)
Definitely able to share	19.4	19.8	19.0
Probably able to share	41.4	38.0	44.8
Not sure	20.4	19.0	21.8
Probably not able to share	12.4	15.6	9.2
Definitely not able to share	6.4	7.6	5.2

For peer review only - http://bmjopen.bmj.com/site/about/guidelines.xhtml

Supplementary material 2 - KCL Disaster Data Protection Topline Results for Wave 2

8. A law firm, so they can offer to represent you in a 'no-win, no-fee' claim for compensation

	% (TOTAL – Base = 1000)	% (SAMPLE A – Base = 500)	% (SAMPLE B – Base = 500)
Definitely able to share	3.8	5.0	2.6
Probably able to share	9.0	8.4	9.6
Not sure	17.9	16.0	19.8
Probably not able to share	25.1	26.2	24.0
Definitely not able to share	44.2	44.4	44.0

Q6. Following an incident like this, various groups or individuals might ask the police to tell them who was caught up in the incident. For each of the following groups, please say whether you think that is acceptable or not acceptable for the police to share your name and contact details with these groups or people following an incident like this, without checking with you first;

1. Your GP, so they can check how you are doing

	% (TOTAL – Base = 1000)	% (SAMPLE A – Base = 500)	% (SAMPLE B – Base = 500)
Definitely acceptable to share	30.1	28.6	31.6
Probably acceptable to share	41.0	41.6	40.4
Not sure	13.0	12.0	14.0
Probably not acceptable to share	8.0	8.4	7.6
Definitely not acceptable to share	7.9	9.4	6.4

2. Your travel insurance company, so they can offer you practical and financial support

	% (TOTAL – Base = 1000)	% (SAMPLE A – Base = 500)	% (SAMPLE B – Base = 500)
Definitely acceptable to share	10.8	12.2	9.4
Probably acceptable to share	28.8	28.0	29.6
Not sure	20.4	20.0	20.8
Probably not acceptable to share	17.7	19.4	16.0
Definitely not acceptable to share	22.3	20.4	24.2

3. A team of medical researchers from a university, so they can invite you to take part in a study to improve the way future incidents are dealt with

	% (TOTAL – Base = 1000)	% (SAMPLE A – Base = 500)	% (SAMPLE B – Base = 500)
Definitely acceptable to share	4.9	3.4	6.4
Probably acceptable to share	17.7	16.2	19.2
Not sure	24.5	22.4	26.6
Probably not acceptable to share	26.7	30.2	23.2
Definitely not acceptable to share	26.2	27.8	24.6

4. A journalist, so they can write a news article about the incident

	% (TOTAL – Base = 1000)	% (SAMPLE A – Base = 500)	% (SAMPLE B – Base = 500)
Definitely acceptable to share	1.8	2.0	1.6
Probably acceptable to share	4.8	4.2	5.4
Not sure	10.1	7.4	12.8
Probably not acceptable to share	15.6	15.4	15.8
Definitely not acceptable to share	67.7	71.0	64.4

For peer review only - http://bmjopen.bmj.com/site/about/guidelines.xhtml

Supplementary material 2 - KCL Disaster Data Protection Topline Results for Wave 2

5. A charity, such as the British Red Cross, so they can offer you support

	% (TOTAL – Base = 1000)	% (SAMPLE A – Base = 500)	% (SAMPLE B – Base = 500)
Definitely acceptable to share	7.2	7.0	7.4
Probably acceptable to share	29.8	28.8	30.8
Not sure	25.1	25.4	24.8
Probably not acceptable to share	20.2	21.6	18.8
Definitely not acceptable to share	17.7	17.2	18.2

6. A health-related Government organisation, so they can send you a questionnaire to find out if you might benefit from extra care or support.

	% (TOTAL – Base = 1000)	% (SAMPLE A – Base = 500)	% (SAMPLE B – Base = 500)
Definitely acceptable to share	12.8	11.8	13.8
Probably acceptable to share	39.2	39.0	39.4
Not sure	21.2	20.6	21.8
Probably not acceptable to share	13.8	15.2	12.4
Definitely not acceptable to share	13.0	13.4	12.6

7. A specialist NHS team, to provide you with information about ways to get support for any physical or mental health issues.

	% (TOTAL – Base = 1000)	% (SAMPLE A – Base = 500)	% (SAMPLE B – Base = 500)
Definitely acceptable to share	28.1	28.2	28.0
Probably acceptable to share	44.6	44.6	44.6
Not sure	12.5	11.4	13.6
Probably not acceptable to share	7.8	8.2	7.4
Definitely not acceptable to share	7.0	7.6	6.4

8. A law firm, so they can offer to represent you in a 'no-win, no-fee' claim for compensation

	% (TOTAL – Base = 1000)	% (SAMPLE A – Base = 500)	% (SAMPLE B – Base = 500)
Definitely acceptable to share	2.7	3.6	1.8
Probably acceptable to share	7.8	7.2	8.4
Not sure	13.8	13.2	14.4
Probably not acceptable to share	19.7	19.4	20.0
Definitely not acceptable to share	56.0	56.6	55.4

Following a terrorist incident, people sometimes develop mental health problems as a result of their experiences. To provide help, the Government sometimes sets up a specialist NHS service to offer support to those who need it. A government organisation would be asked to help with this by putting together a confidential database of people who were present and then writing to them with information about the NHS service and a short mental health questionnaire.

Q7. To what extent do you agree or disagree with the following statement. If I was caught up in a terrorist incident, I would want the police to give my contact details to the government organisation so that they can contact me about this service.

	%
Strongly agree	21.1
Tend to agree	45.8
Neither agree nor disagree	17.5
Tend to disagree	9.7
Strongly disagree	5.9

For peer review only - http://bmjopen.bmj.com/site/about/guidelines.xhtml

Supplementary material 2 - KCL Disaster Data Protection Topline Results for Wave 2

Q8. And why do you say that? OPEN-ENDED RESPONSES

Q9. To what extent do you agree or disagree with the following statement: If I was caught up in a terrorist incident, I would want my name to be included on this database.

	%
Strongly agree	13.5
Tend to agree	36.6
Neither agree nor disagree	26.5
Tend to disagree	14.9
Strongly disagree	8.5

Q10. To what extent do you agree or disagree with the following statement: If I was caught up in a terrorist incident, I would be unhappy if my name was included on this database.

	%
Strongly agree	10.2
Tend to agree	18.5
Neither agree nor disagree	28.0
Tend to disagree	32.1
Strongly disagree	11.2

Q11A. Imagine the scenario we described occurred and the police decided to share your name and contact information with a health-related government organisation. Please say how much you agree or disagree with the following statements:

1. **I would be concerned that my details would be made public by accident**

	% (TOTAL – Base = 1000)	% (SAMPLE A – Base = 500)	% (SAMPLE B – Base = 500)
Strongly agree	27.0	30.2	23.8
Tend to agree	35.8	34.4	37.2
Neither agree nor disagree	20.7	19.8	21.6
Tend to disagree	13.8	13.4	14.2
Strongly disagree	2.7	2.2	3.2

2. **I would trust the health organisation to keep my details secure**

	% (TOTAL – Base = 1000)	% (SAMPLE A – Base = 500)	% (SAMPLE B – Base = 500)
Strongly agree	14.2	14.8	13.6
Tend to agree	39.9	36.8	43.0
Neither agree nor disagree	25.5	26.6	24.4
Tend to disagree	13.9	15.2	12.6
Strongly disagree	6.5	6.6	6.4

3. **It would be an invasion of my privacy**

	% (TOTAL – Base = 1000)	% (SAMPLE A – Base = 500)	% (SAMPLE B – Base = 500)
Strongly agree	18.6	20.8	16.4
Tend to agree	23.6	25.2	22.0
Neither agree nor disagree	30.1	29.4	30.8
Tend to disagree	23.1	20.2	26.0
Strongly disagree	4.6	4.4	4.8

For peer review only - http://bmjopen.bmj.com/site/about/guidelines.xhtml

Supplementary material 2 - KCL Disaster Data Protection Topline Results for Wave 2

4. I would be concerned about how my information might be used in the future

	% (TOTAL – Base = 1000)	% (SAMPLE A – Base = 500)	% (SAMPLE B – Base = 500)
Strongly agree	28.2	29.8	26.6
Tend to agree	40.3	38.2	42.4
Neither agree nor disagree	18.8	19.0	18.6
Tend to disagree	10.1	10.4	9.8
Strongly disagree	2.6	2.6	2.6

5. I would be concerned that my employer would be told information about me

	% (TOTAL – Base = 1000)	% (SAMPLE A – Base = 500)	% (SAMPLE B – Base = 500)
Strongly agree	11.0	11.6	10.4
Tend to agree	23.1	21.4	24.8
Neither agree nor disagree	29.5	28.2	30.8
Tend to disagree	25.4	27.4	23.4
Strongly disagree	11.0	11.4	10.6

Q11B. Imagine the scenario we described occurred and the police decided to share your name and contact information with a health-related government organisation. Please say how much you agree or disagree with the following statements:

1. I would be concerned that my details might be shared by the health organisation with other groups without my permission

	% (TOTAL – Base = 1000)	% (SAMPLE A – Base = 500)	% (SAMPLE B – Base = 500)
Strongly agree	26.1	27.2	25.0
Tend to agree	38.2	41.0	35.4
Neither agree nor disagree	21.9	18.2	25.6
Tend to disagree	11.5	11.0	12.0
Strongly disagree	2.3	2.6	2.0

2. I would want to be kept informed about how my information was being used

	% (TOTAL – Base = 1000)	% (SAMPLE A – Base = 500)	% (SAMPLE B – Base = 500)
Strongly agree	48.0	49.6	46.4
Tend to agree	35.8	35.6	36.0
Neither agree nor disagree	13.3	12.0	14.6
Tend to disagree	2.1	1.4	2.8
Strongly disagree	0.8	1.4	*

3. It wouldn't bother me at all

	% (TOTAL – Base = 1000)	% (SAMPLE A – Base = 500)	% (SAMPLE B – Base = 500)
Strongly agree	6.6	6.2	7.0
Tend to agree	22.4	21.8	23.0
Neither agree nor disagree	30.6	29.8	31.4
Tend to disagree	25.8	26.4	25.2
Strongly disagree	14.6	15.8	13.4

For peer review only - http://bmjopen.bmj.com/site/about/guidelines.xhtml

Supplementary material 2 - KCL Disaster Data Protection Topline Results for Wave 2

4. I would be reassured that a health organisation was looking out for me

	% (TOTAL – Base = 1000)	% (SAMPLE A – Base = 500)	% (SAMPLE B – Base = 500)
Strongly agree	11.8	10.8	12.8
Tend to agree	43.7	42.0	45.4
Neither agree nor disagree	28.8	28.6	29.0
Tend to disagree	10.0	12.2	7.8
Strongly disagree	5.7	6.4	5.0

5. I would be concerned that my GP might be told

	% (TOTAL – Base = 1000)	% (SAMPLE A – Base = 500)	% (SAMPLE B – Base = 500)
Strongly agree	4.5	4.4	4.6
Tend to agree	10.1	9.6	10.6
Neither agree nor disagree	28.1	29.2	27.0
Tend to disagree	36.9	35.6	38.2
Strongly disagree	20.4	21.2	19.6

Q12. Please state the highest level of education you have achieved:

	%
Left school without qualifications	2.7
Secondary education (O-level/GCSE/A-level)	41.3
Higher education (BSc, BA/higher qualification)	55.5
Prefer not to say	0.5

For peer review only

STROBE Statement—Checklist of items that should be included in reports of ***cross-sectional studies***

	Item No	Recommendation	Page No(s)
**Title and abstract**	1	(*a*) Indicate the study's design with a commonly used term in the title or the abstract	1
		(*b*) Provide in the abstract an informative and balanced summary of what was done and what was found	2
**Introduction**			
Background/ rationale	2	Explain the scientific background and rationale for the investigation being reported	4
Objectives	3	State specific objectives, including any prespecified hypotheses	5
**Methods**			
Study design	4	Present key elements of study design early in the paper	6
Setting	5	Describe the setting, locations, and relevant dates, including periods of recruitment, exposure, follow-up, and data collection	6
Participants	6	(*a*) Give the eligibility criteria, and the sources and methods of selection of participants	6
Variables	7	Clearly define all outcomes, exposures, predictors, potential confounders, and effect modifiers. Give diagnostic criteria, if applicable	7-8
Data sources/ measurement	8*	For each variable of interest, give sources of data and details of methods of assessment (measurement). Describe comparability of assessment methods if there is more than one group	7-8
Bias	9	Describe any efforts to address potential sources of bias	6
Study size	10	Explain how the study size was arrived at	6
Quantitative variables	11	Explain how quantitative variables were handled in the analyses. If applicable, describe which groupings were chosen and why	8
Statistical methods	12	(*a*) Describe all statistical methods, including those used to control for confounding	8
		(*b*) Describe any methods used to examine subgroups and interactions	N/A
		(*c*) Explain how missing data were addressed	N/A
		(*d*) If applicable, describe analytical methods taking account of sampling strategy	N/A
		(*e*) Describe any sensitivity analyses	N/A
**Results**			
Participants	13*	(a) Report numbers of individuals at each stage of study—eg numbers potentially eligible, examined for eligibility, confirmed eligible, included in the study, completing follow-up, and analysed	8
		(b) Give reasons for non-participation at each stage	8
		(c) Consider use of a flow diagram	
Descriptive data	14*	(a) Give characteristics of study participants (eg demographic, clinical, social) and information on exposures and potential confounders	Table 1
		(b) Indicate number of participants with missing data for each variable of interest	N/A
Outcome data	15*	Report numbers of outcome events or summary measures	9-10
Main results	16	(*a*) Give unadjusted estimates and, if applicable, confounder-adjusted estimates and their precision (eg, 95% confidence interval). Make clear which confounders were adjusted for and why they were included	9-10
		(*b*) Report category boundaries when continuous variables were categorized	Tables 3-5
		(*c*) If relevant, consider translating estimates of relative risk into absolute risk for a meaningful time period	N/A
Other analyses	17	Report other analyses done—eg analyses of subgroups and interactions, and sensitivity analyses	N/A
**Discussion**			
Key results	18	Summarise key results with reference to study objectives	11
Limitations	19	Discuss limitations of the study, taking into account sources of potential bias or imprecision. Discuss both direction and magnitude of	12

For peer review only

		any potential bias	
Interpretation	20	Give a cautious overall interpretation of results considering objectives, limitations, multiplicity of analyses, results from similar studies, and other relevant evidence	13
Generalisability	21	Discuss the generalisability (external validity) of the study results	12-13
**Other information**			
Funding	22	Give the source of funding and the role of the funders for the present study and, if applicable, for the original study on which the present article is based	14

*Give information separately for exposed and unexposed groups.

Note: An Explanation and Elaboration article discusses each checklist item and gives methodological background and published examples of transparent reporting. The STROBE checklist is best used in conjunction with this article (freely available on the Web sites of PLoS Medicine at http://www.plosmedicine.org/, Annals of Internal Medicine at http://www.annals.org/, and Epidemiology at http://www.epidem.com/). Information on the STROBE Initiative is available at www.strobe-statement.org.

For peer review only

BMJ Open

Public attitudes in England towards the sharing of personal data following a mass casualty incident: A cross-sectional study

Journal:	*BMJ Open*
Manuscript ID	bmjopen-2018-022852.R1
Article Type:	Research
Date Submitted by the Author:	23-Apr-2018
Complete List of Authors:	Rubin, GJ; King's College London, Psychological Medicine<br>Webster, Rebecca; King's College London, Psychological Medicine<br>Rubin, Antonia<br>Amlot, Richard; Public Health England, Behavioural Science Emergency Response Department Science and Technology<br>Grey, N; Sussex Partnership NHS Foundation Trust<br>greenberg, neil; Kings College London, Kings Centre for Military Health Research
**Primary Subject Heading**:	Public health
Secondary Subject Heading:	Emergency medicine
Keywords:	Data sharing, Mass casualty incident, Public attitudes, Terrorism, Shooting, Radiation

SCHOLARONE™
Manuscripts

Public attitudes in England towards the sharing of personal data following a mass casualty incident: A cross-sectional study

G James Rubin, Rebecca Webster, Antonia N Rubin, Richard Amlôt, Nick Grey, Neil Greenberg

G James Rubin, Reader in the Psychology of Emerging Health Risks, Department of Psychological Medicine, King's College London

Rebecca Webster, PhD student, Department of Psychological Medicine, King's College London

Antonia N Rubin, Lawyer, Tonbridge, Kent.

Richard Amlôt, Scientific Programme Leader, Behavioural Science Emergency Response Department Science and Technology (ERD S&T), Health Protection Directorate, Porton Down, Salisbury, Public Health England

Nick Grey, Consultant Clinical Psychologist, Aldrington House, Sussex Partnership NHS Foundation Trust, 35 New Church Road, Hove, BN3 4AG, and University of Sussex

Neil Greenberg, Professor of Defence Mental Health, Department of Psychological Medicine, King's College London

Correspondence to: Gideon.rubin@kcl.ac.uk

For peer review only

Abstract

Objectives: To assess public attitudes towards data sharing to facilitate a mental health screening programme for people caught up in a mass casualty incident.

Design: Two, identical, cross-sectional, on-line surveys, using quotas to ensure demographic representativeness of 18 to 65 year olds in England. Participants were randomly allocated to consider a scenario in which they witness a terrorism-related radiation incident or mass shooting, after which a police officer records their contact details.

Setting: Participants were drawn from an on-line panel maintained by a market research company. Surveys were conducted before and immediately after a series of terrorist attacks and a large tower block fire occurred in England.

Participants: One thousand people aged 18 to 65 years participated in each survey.

Main outcome measures: Three questions asking participants if it would acceptable for police to share their contact details, without asking first, with "a health-related government organisation, so they can send you a questionnaire to find out if you might benefit from extra care or support," "a specialist NHS team, to provide you with information about ways to get support for any physical or mental health issues," and "your GP, so they can check how you are doing."

Results: A minority of participants reported that it would be definitely not acceptable for their details to be shared with the government organisation (n=259, 13.0%), the NHS (n=141, 7.1%) and their GP (n=166, 8.3%). There was a small, but significant increase in acceptability for the radiation incident compared to the mass shooting. No major differences were observed between the pre-incidents and post-incidents surveys.

Conclusions: Although most people believe it is acceptable for their details to be shared in order to facilitate a mental health response to a major incident, care must be taken to communicate with those affected about how their information will be used.

For peer review only

For peer review only - http://bmjopen.bmj.com/site/about/guidelines.xhtml

Strengths and limitations

- The survey sample was demographically representative of 18 to 65 year olds in England.
- The survey was conducted again after the recent terrorist incidents and the Grenfell tower fire as we felt that the scenarios may have become more salient for respondents, providing a better test of attitudes towards data sharing in such contexts.
- Although demographically representative, our sample volunteered to answer internet surveys and may be more accepting of data sharing than those who did not volunteer.
- The scenarios used in our surveys were hypothetical. Being directly involved in a major incident may alter someone's views about the desirability of data sharing.
- Participants were asked to consider that neither they, nor anyone they knew well, had been harmed in the described scenarios. This may have reduced the perceived need to participate in a psychological screening programme and hence the perceived acceptability of data sharing.

For peer review only

For peer review only - http://bmjopen.bmj.com/site/about/guidelines.xhtml

Introduction

Following a disaster, terrorist attack or other mass casualty incident, rates of mental distress and disorder among survivors, their relatives, eyewitnesses and first responders can be substantial (1). Although evidence-based interventions are available to treat established mental health disorder, failure to seek professional help for mental illness is common as a result of stigma and other barriers to care (2, 3). Because of this, in their guidance on the detection and treatment of post-traumatic stress disorder, the UK National Institute for Health and Clinical Excellence recommends that, "screening of all [affected] individuals should be considered by the authorities responsible for developing the local disaster plan."(4) Within the UK the first use of a 'screen and treat' approach occurred following the 7 July 2005 bombings in London. A central team used a range of methods to contact as many people as possible who had been caught up in the incident. Those contacted were asked to complete a short mental health questionnaire, invited to have a more detailed assessment if need-be, and referred for treatment where required (5). Although the process resulted in many patients receiving treatment who might not otherwise have done so, difficulties were encountered in assembling a comprehensive list of people to be screened. Sharing of information between agencies was hampered by a cautious interpretation of legislation designed to safeguard personal data (6). As a result, only 910 people were contacted out of an estimated 4,000 who were exposed to the attacks. Within the UK, greater clarity about the flexibility that responders have to share information was subsequently provided (6). This guidance notes that where information is not "sensitive" (i.e. related to ethnicity, political opinion, religious belief, trade union membership, health, sexual life or criminal activity) then it is acceptable for names and contact details to be shared between official agencies to facilitate the provision of care to those affected.

In June 2015, 38 people were killed by a terrorist in Sousse, Tunisia. Thirty of those killed were British holidaymakers. Given the large number of British tourists who witnessed the attacks and felt their own lives to be in danger, a psychological health screening programme was again set up and subsequently widened to include victims of terrorist attacks in Brussels and Paris. Public Health England was given the task of setting up a registry of all English nationals who were directly affected by the attacks, something which might also be considered in the future for other forms of disaster(7). Once again, however, obtaining the contact details for many of those affected was not possible. Although the police collected contact details from holiday makers as they returned to England, legal advice prevented them from sharing this information. Difficulty in sharing information between agencies often hampers response efforts following a major incident. While it is sometimes possible to find more indirect routes to contact people, these approaches are often less than ideal. For example, while it might be possible for the police to send out information to people on behalf of a screening service or to ask for permission before sharing data, in practice, such strategies can be time consuming to agree, often result in a poorer response rate and prevent any single agency from

For peer review only - http://bmjopen.bmj.com/site/about/guidelines.xhtml

combining overlapping lists of names held by different groups into a single, consolidated list of those affected.

Clearly, uncertainties still exist about when it is appropriate to share information following a major incident. Changes to legislation in 2018 will create stiffer penalties for organisations found to be in breach of data protection law and are likely to create an even more cautious approach to data sharing. But how members of the public themselves view this matter is unclear. Recent polling suggests that public attitudes towards data sharing are shaped by who is doing the sharing, what for, and whether safeguards are in place (8). Data sharing for the public good is generally viewed positively. For example, only 9% of respondents to one large UK survey felt that it would be an invasion of privacy if details held about them on a cancer registry were used to invite them to participate in a study conducted by a university medical school (9). Where a personal benefit is possible, people also tend to be more willing for information to be shared (10). To our knowledge there has been no research on the public acceptability of data sharing in the context of a major public health incident, disaster or terrorist attack.

In this study, we used an on-line quota survey of a sample of adults in England designed to reflect the known population profile, to identify public attitudes towards the sharing of personal information following a mass casualty incident and the setting up of a register of affected people. We tested whether attitudes might differ depending on whether the incident resulted primarily in a mental or physical health risk for those involved in the incident. Soon after we conducted our survey, four terrorist attacks and a tower block fire occurred in England killing over a hundred people. Given the intense media attention about the need to provide support to victims of these incidents, we felt that the scenarios described in our survey may have become more salient for respondents, providing a better test of attitudes towards data sharing in such contexts. We therefore repeated the survey with a new sample of respondents.

Method

Design

We commissioned the market research organisation Ipsos MORI to conduct two on-line surveys of 1,000 adult (18 to 65) residents of England. Data collection for the initial, pre-incidents survey took place between 9 and 15 March 2017. Four terrorist attacks then occurred in England between 22 March and 19 June, killing 34, and a tower block fire occurred on 14 June, killing 71. Data collection for our second, post-incidents survey took place between 3 and 10 July 2017. The procedures and questionnaires for both surveys were identical. The study was approved by the Psychiatry, Nursing and Midwifery Research Ethics Committee at King’s College London (ref: HR-16/17-3814).

For peer review only - http://bmjopen.bmj.com/site/about/guidelines.xhtml

Participants

Ipsos MORI recruited participants from an existing panel of people willing to take part in internet surveys (approximate N=160,000). Panel participants typically receive points for every survey they complete: for our surveys, participants received points equivalent to 30 pence. Quotas based on participant age and gender (interlocked), location, and working status were used to ensure that the sample was demographically representative of adults aged 18-65 in England, according to data from the National Readership Survey (11). We excluded adults aged over 65 on the basis that older adults who are members of an internet survey panel may not be representative of the general population of older adults (12). We intended to recruit 1,000 participants for each survey to provide us with a maximum sample error of about plus or minus 3%.

Scenarios

Ipsos MORI emailed a link to the survey to potential participants. After providing informed consent and clicking through to begin the survey, participants received questions about one of two hypothetical scenarios. Which scenario they received was decided by the survey software, based on which scenario had the lowest number of completed responses at that time. We based the scenarios on an example given in HM Government's 'Data Protection and Sharing – Guidance for Emergency Planners and Responders' (6) (case study 5). The two versions represented an incident that might primarily pose a mental health threat (witnessing shooting) or a physical health threat (exposure to radiation). In the first version, participants were asked to, "imagine that you are on holiday in another country and witness a terrorist shooting. Some people are badly injured but you are not harmed and neither is anyone you know well." The second version asked them to, "imagine that you are on holiday in another country and a place you visit is discovered by police to be contaminated by radioactive material. The police believe this is linked to a terrorist group. Some people are badly injured but you are not harmed and neither is anyone you know well." Participants in both versions were then told that "the British government arranges for you to be flown home, along with other British nationals who were in the area. When you arrive back in Britain, a police officer at the airport records your name, address, phone number and email address. The following questions ask about things that might happen next."

Questionnaire

The supplementary materials show the full text for all items. We first presented participants with eight groups who might ask the police to share information with them following the incident, together with their reasons for this. In each case we asked whether the participant thought the police currently could share information with this group without asking the participant first, and whether the participant thought it was acceptable for the police to share their information with the group.

For peer review only - http://bmjopen.bmj.com/site/about/guidelines.xhtml

We next provided information about the rationale and process for a mental health screen and treat programme. We described it as a specialist NHS service set up to offer support and treatment to those who need it and explained that a government organisation would support the service by putting together a confidential database of those affected and writing to them with a screening questionnaire. We asked participants to state their level of agreement with the statements that, "If I was caught up in a terrorist incident, I would want the police to give my contact details to the government organisation so that they can contact me about this service;" "I would want my name to be included on this database;" and "I would be unhappy if my name was included on this database."

Participants were then asked to reconsider the scenario they had been presented with and told that the police had decided to share their name and contact details with a health-related government organisation. They were asked to state their agreement with 10 statements concerning their views about this (e.g. "it would be an invasion of my privacy").

We asked all participants about their age, gender, working status, where in England they lived and their highest educational qualification.

Analysis

We calculated the proportion of respondents endorsing each response option and assessed the difference in responses between the pre- and post-incidents surveys and between the two scenarios using chi-square tests.

Patient Involvement

No patients or members of the public were involved in the design, recruitment or conduct of the study. Due to anonymity, results cannot be disseminated to study participants unless they specifically request them from the researcher.

Results

The topline results provided by Ipsos MORI for both surveys can be found in supplementary file 1 and supplementary file 2.

Response rates and demographics

Response rates to the invitation emails were 10% (pre-incident) and 6% (post-incident). These are normal rates for surveys of this nature. The lower response rate for the post-incident survey may reflect the fact that data collection occurred during July, which coincides with the Summer holiday period in England. In each case, a small proportion of respondents (45 in total) were excluded from

For peer review only - http://bmjopen.bmj.com/site/about/guidelines.xhtml

the data for completing the surveys suspiciously quickly or giving illogically identical answers to multiple consecutive questions. Demographics are shown in Table 1.

Perceived ability and acceptability of the police sharing data with other agencies

Table 2 presents data on the perceived ability of the police to share personal data with other agencies. The option that was most commonly believed to be within the police's current powers was sharing data with "a specialist NHS team, to provide you with information about ways to get support for any physical or mental health issues" (endorsed by 60.8% overall). 53.9% of respondents believed that police could definitely or probably share data with "your GP, so they can check how you are doing," while 45.9% believed that data could definitely or probably be shared with "a health-related government organisation, so they can send you a questionnaire to find out if you might benefit from extra care or support." No differences were found between the pre- and post-incident surveys. Significantly more people believed that data could be shared following a radiation incident compared to a shooting incident with: your GP; a health-related government organisation; and a specialist NHS team.

Table 3 presents data on the acceptability of the police sharing personal data. The option that was most commonly seen as being acceptable was sharing data with a specialist NHS team (seen as definitely acceptable by 27.6% and probably acceptable by 44.3%). Most respondents believed it was definitely (29.9%) or probably (40.2%) acceptable for the police to share data with their GP, while a smaller number believed it was definitely (13.0%) or probably (37.6%) acceptable for data to be shared with a health-related government organisation. No differences were found between the pre- and post-incident surveys, aside from a small reduction in the post-incident data in the acceptability of information being shared with medical researchers. Significantly more people believed it would be acceptable to share their data following a radiation incident compared to a shooting incident with: their GP; a team of university medical researchers; and a health-related government organisation.

Attitudes towards data sharing to enable a screen and treat programme or database

Table 4 presents the attitudes of participants towards a screen and treat programme and confidential database. Although most respondents strongly agreed (21.0%) or tended to agree (45.6%) that they would want the police to share their contact details to allow them to be contacted about screening, 10.7% tended to disagree and 5.2% strongly disagreed. In total, 51.6% strongly or tended to agree with wanting their name to appear on the database, while 18.3% tended to agree that they would be unhappy if it was included on the database and 9.6% strongly agreed that they would be unhappy. No differences were found between the pre- and post-incident surveys. Because these questions asked about "a terrorist incident" in general, we did not test for differences between the shooting and radiation scenarios.

For peer review only - http://bmjopen.bmj.com/site/about/guidelines.xhtml

Table 5 presents the broader attitudes of participants towards data-sharing in the context of the radiation and shooting scenarios. Concerns endorsed by more than 50% of respondents about the police sharing their information were that: my details would be made public by accident; I would be concerned about how my information might be used in the future; and I would be concerned that my details might be shared by the health organisation with other groups without my permission. Most participants reported wanting to be kept informed about how their information was being used. More positively, 55.4% strongly agreed or tended to agree that they would be "reassured that a health organisation was looking out for me" and 54.4% would trust the health organisation to keep their details secure. No differences were found between and pre- and post-incidents surveys. Significantly more people in the radiation than the shooting scenario reported that they would be reassured that a health organisation was looking out for them and also that they would want to be kept informed about how their information was being used. Significantly fewer people in the radiation scenario reported that the sharing would be an invasion of their privacy.

For peer review only

For peer review only - http://bmjopen.bmj.com/site/about/guidelines.xhtml

Discussion

Our data suggest that not only do the majority of people believe that it is acceptable for the police to share their details with health agencies in the aftermath of a major incident, but that most people believe that the police are currently able to do this under existing legislation. This belief is not simply due to a broader misunderstanding among the public about their data protection rights: only 8% believe that the police are able to share data with journalists, for example. Instead, in line with findings reported by others (10), it appears that where a direct benefit to the individual might result from sharing information with a trusted source such as a GP or NHS service, the public have a greater acceptability of it, and an expectation that it will occur. However, such views were not unanimous among our participants. It was notable that 7% of our sample thought it was definitely not acceptable for their details to be shared with an NHS service following a major incident, 8% thought it was definitely not acceptable for their details to be shared with their GP, and 10% strongly agreed that they would be unhappy for their name to be included on a confidential database of those affected. More people (8%, 9% and 18%, respectively) thought these actions were probably not acceptable or tended to agree that they would be unhappy. These figures must be taken into consideration by responding agencies. Given that it is impossible to know in advance who is or is not willing for their data to be shared, not sharing data without prior permission to protect the interests of this minority may be a justifiable position in some cases.

Our data provide some indications as to why people may be concerned about data sharing following an incident. In particular, the risk of accidental or deliberate sharing of information to a third party was noted as a concern by many. If data sharing or the setting up of a database is to be considered following any future incident, a robust policy around further sharing should be developed and explained to those affected. Keeping people regularly updated on how their data are being used is also important. More generally, the nature of the incident itself is a determinant of the acceptability of data sharing and the use of a database. Participants asked to imagine a scenario that might pose a long-term physical health risk to themselves (possible exposure to radiation) were more likely to have a positive view of data sharing than those asked to consider a risk to their mental health (witnessing a shooting). Whether this was because participants found it hard to envisage that their mental health might be affected in the scenarios that we used, or whether this reflects the broader stigma associated with mental illness is unclear. Similarly, while people may be familiar with the concept of physical health effects that develop some time later after a radiation incident, they may be less familiar with the concept of mental health effects that only become apparent later: this lack of understanding may also have reduced the perceived desirability of a mental health screening programme across both conditions. It is important also not to overstate the differences that we observed, however. Although statistically significant, in many cases these were small. Nonetheless, additional care might be required when communicating with the public about data sharing that is primarily intended to support

For peer review only - http://bmjopen.bmj.com/site/about/guidelines.xhtml

a mental health response. It is possible that ongoing public campaigns to improve the understanding of mental ill-health may impact upon these differences over time.

Limitations

Several limitations with our data need to be considered. First, we restricted our sample to people aged 18 to 65. We are unable to extrapolate from our data to those older or younger than this. Second, although our sample was demographically representative of 18 to 65 year olds in England, it may not have been psychologically representative of this population. In particular, it seems plausible that people who volunteer to answer internet surveys may be more accepting of data sharing than those who do not volunteer. If anything, our estimate of the number of people expressing reticence about data sharing is therefore likely to be an underestimate. Third, the scenarios used in our surveys were hypothetical. We hoped that repeating our survey soon after a series of terrorist attacks and a major disaster would make the issues involved more salient for participants. Doing this did not alter our results, possibly reflecting the fact that participants in our first survey were already thinking carefully about the scenarios that we presented. Nonetheless, it is possible that being directly involved in a major incident would alter someone's views about the desirability of data sharing. Fourth, for ethical reasons, we asked participants to consider that neither they, nor anyone they knew well, had been harmed in the scenarios that we described. This may have reduced the perceived need to participate in a psychological screening programme and hence the perceived acceptability of data sharing. People who are injured, bereaved or otherwise harmed in an incident may be more willing for their data to be shared. Finally, we have no information on our participants' previous experiences of data sharing or data protection breaches. Prior experience of breaches, from whatever source or agency, may make people more reluctant to consider future data sharing. This may be relevant for the minority of people in our sample who were reluctant to share data. It would also reinforce the need for clear information and reassurance about data handling.

Conclusion

Disagreement often exists between officials and agencies about the appropriateness of, and best mechanisms for, data sharing in the aftermath of a major incident. These disagreements are also apparent among members of the public: while most support the sharing of data between the police and health services in order to facilitate public health measures, this is by no means a unanimous view. The absence of a clear consensus reinforces the view that agencies should consider the benefits and risks of sharing data with organisations that seek to protect their mental health. In order to assuage public concerns, agencies who hold such data should communicate to the public their policies for securely holding the data and for updating people on how the data will be used.

Author contributions

For peer review only - http://bmjopen.bmj.com/site/about/guidelines.xhtml

GJR developed the idea for the survey and the survey materials in conjunction with ANR, RA, NG and NG. RW carried out the data analysis. GJR drafted the manuscript and revised the manuscript with comments from all authors. GJR accepts full responsibility for the work, had access to the data and controlled the decision to publish.

Financial disclosure statement

The research was funded by the National Institute for Health Research Health Protection Research Unit (NIHR HPRU) in Emergency Preparedness and Response at King's College London in partnership with Public Health England (PHE), and by the NIHR HPRU in Evaluation of Interventions at the University of Bristol in partnership with PHE. The views expressed are those of the author(s) and not necessarily those of the NHS, the NIHR, the Department of Health or Public Health England.

Copyright statement

The Corresponding Author has the right to grant on behalf of all authors and does grant on behalf of all authors, a worldwide licence to the Publishers and its licensees in perpetuity, in all forms, formats and media (whether known now or created in the future), to i) publish, reproduce, distribute, display and store the Contribution, ii) translate the Contribution into other languages, create adaptations, reprints, include within collections and create summaries, extracts and/or, abstracts of the Contribution, iii) create any other derivative work(s) based on the Contribution, iv) to exploit all subsidiary rights in the Contribution, v) the inclusion of electronic links from the Contribution to third party material where-ever it may be located; and, vi) licence any third party to do any or all of the above.

Declaration of competing interests

Competing interests: All authors have completed the ICMJE uniform disclosure form at www.icmje.org/coi_disclosure.pdf and declare: support from the NIHR HPRUs in Emergency Preparedness and Response and in Evaluation of Interventions for the submitted work; no financial relationships with any organisations that might have an interest in the submitted work in the previous three years; no other relationships or activities that could appear to have influenced the submitted work.

Full access to data

All authors had full access to all of the data in the study and can take responsibility for the integrity of the data and the accuracy of the data analysis.

Ethics approval

For peer review only - http://bmjopen.bmj.com/site/about/guidelines.xhtml

The study was approved by the Psychiatry, Nursing and Midwifery Research Ethics Committee at King's College London (ref: HR-16/17-3814). All participants provided informed consent before starting the surveys.

Transparency declaration

The lead author confirms that this manuscript is an honest, accurate and transparent account of the study being reported. No important aspects of the study have been omitted and any discrepancies from the study as planned have been explained.

Data sharing

No additional data available.

For peer review only

References

1. Whalley MG, Brewin CR. Mental health following terrorist attacks. British Journal of Psychiatry. 2007;190(FEB.):94-6.

2. Stuber J, Galea S, Boscarino JA, Schlesinger M. Was there unmet mental health need after the September 11, 2001 terrorist attacks? Social Psychiatry and Psychiatric Epidemiology. 2006;41(3):230-40.

3. Borschmann R, Greenberg N, Jones N, Henderson RC. Campaigns to reduce mental illness stigma in Europe: A scoping review. Die Psychiatrie. 2014;11(1):43-50.

4. Excellence NIfHaC. Post-traumatic stress disorder: management. Manchester: National Institute for Health and Clinical Excellence; 2005.

5. Brewin CR, Fuchkan N, Huntley Z, Robertson M, Thompson M, Scragg P, et al. Outreach and screening following the 2005 London bombings: Usage and outcomes. Psychological Medicine. 2010;40(12):2049-57.

6. HMGovernment. Data protection and sharing - guidance for emergency planners and responders. London: Cabinet Office; 2007.

7. Close RM, Maguire H, Etherington G, Brewin CR, Fong K, Saliba V, et al. Preparedness for a major incident: Creation of an epidemiology protocol for a health protection register in England. Environment International. 2014;72:75-82.

8. Ipsos MORI. Public attitudes to the use and sharing of their data. Research for the Royal Statistical Society by Ipsos MORI. London: Ipsos MORI; 2014.

9. Barrett G, Cassell JA, Peacock JL, Coleman MP, National Cancer R. National survey of British public's views on use of identifiable medical data by the National Cancer Registry. Bmj. 2006;332(7549):1068-72.

10. Kaufman DJ, Murphy-Bollinger J, Scott J, Hudson KL. Public opinion about the importance of privacy in biobank research. American journal of human genetics. 2009;85(5):643-54.

11. National Readership Survey. National Readership Survey 2015. 2015.

12. Office for National Statistics. Internet users: 2015. Available from: https://www.ons.gov.uk/businessindustryandtrade/itandinternetindustry/bulletins/internetusers/2015 2015.

For peer review only

For peer review only - http://bmjopen.bmj.com/site/about/guidelines.xhtml

Table 1. Demographic characteristics of survey respondents

		Frequency (%)		
**Variable**	**Categories**	**Pre-incidents**	**Post-incidents**	**Total**
Sex	Male	490 (49.0)	496 (49.6)	986 (49.3)
	Female	510 (51.0)	504 (50.4)	1014 (50.7)
Age	18 to 24	119 (11.9)	144 (14.4)	263 (13.2)
	25 to 34	225 (22.5)	222 (22.2)	447 (22.4)
	35 to 44	213 (21.3)	207 (20.7)	420 (21.0)
	45 to 54	233 (23.3)	226 (22.6)	459 (23.0)
	55 to 65	210 (21.0)	201 (20.1)	411 (20.6)
Region	North East	46 (4.6)	46 (4.6)	92 (4.6)
	North West	137 (13.7)	135 (13.5)	272 (13.6)
	Yorkshire and Humberside	98 (9.8)	101 (10.1)	199 (10.0)
	West Midlands	97 (9.7)	101 (10.1)	198 (9.9)
	East Midlands	89 (8.9)	84 (8.4)	173 (8.7)
	East of England	90 (9.0)	99 (9.9)	189 (9.5)
	South West	95 (9.5)	90 (9.0)	185 (9.3)
	South East	167 (16.7)	166 (16.6)	333 (16.7)
	London	181 (18.1)	178 (17.8)	359 (18.0)
Employment status	Working full time	501 (50.1)	509 (50.9)	1010 (50.5)
	Working part time	151 (15.1)	140 (14.0)	291 (14.6)
	Self-employed	70 (7.0)	78 (7.8)	148 (7.4)
	Unemployed, looking for a job	49 (4.9)	52 (5.2)	101 (5.1)
	Unemployed not looking for a job	105 (10.5)	103 (10.3)	208 (10.4)
	Retired	78 (7.8)	56 (5.6)	134 (6.7)
	Pupil / student / in full-time education	46 (4.6)	62 (6.2)	108 (5.4)
Highest level of education achieved	Left school without qualifications	33 (3.3)	27 (2.7)	60 (3.0)
	Secondary education	424 (42.4)	413 (41.3)	837 (41.9)
	Higher education	538 (53.8)	555 (55.5)	1093 (54.7)
	Prefer not to say	5 (0.5)	5 (0.5)	10 (0.5)

For peer review only - http://bmjopen.bmj.com/site/about/guidelines.xhtml

Table 2. Ability of police sharing personal data with other agencies, without asking first

Agency requesting personal information and reason for the request		Frequency (%) believing if police are able to share for this reason						
		Shooting	Radiation	χ^2	Pre-incidents	Post-incidents	χ^2	Total
Your GP, so they can check how you are doing	Definitely able	169 (16.9)	208 (20.8)		181 (18.1)	196 (19.6)		377 (18.9)
	Probably able	328 (32.8)	373 (37.3)		360 (36.0)	341 (34.1)		701 (35.1)
	Not sure	212 (21.2)	203 (20.3)	20.521***	206 (20.6)	209 (20.9)	1.191	415 (20.8)
	Probably not able	184 (18.4)	122 (12.2)		154 (15.4)	152 (15.2)		306 (15.3)
	Definitely not able	107 (10.7)	94 (9.4)		99 (9.9)	102 (10.2)		201 (10.1)
Your travel insurance company, so they can offer you practical and financial support	Definitely able	97 (9.7)	93 (9.3)		84 (8.4)	106 (10.6)		190 (9.5)
	Probably able	235 (23.5)	230 (23.0)		236 (23.6)	229 (22.9)		465 (23.3)
	Not sure	254 (25.4)	263 (26.3)	1.553	271 (27.1)	246 (24.6)	4.440	517 (25.9)
	Probably not able	242 (24.2)	226 (22.6)		236 (23.6)	232 (23.2)		468 (23.4)
	Definitely not able	172 (17.2)	188 (18.8)		173 (17.3)	187 (18.7)		360 (18.0)
A team of medical researchers from a university, so they can invite you to take part in a study to improve the way future incidents are dealt with	Definitely able	38 (3.8)	57 (5.7)		51 (5.1)	44 (4.4)		95 (4.8)
	Probably able	123 (12.3)	144 (14.4)		135 (13.5)	132 (13.2)		267 (13.3)
	Not sure	226 (22.6)	232 (23.3)	7.437	221 (22.1)	237 (23.7)	1.220	458 (22.9)
	Probably not able	314 (31.4)	296 (29.6)		309 (30.9)	301 (30.1)		610 (30.5)
	Definitely not able	299 (29.9)	271 (27.1)		284 (28.4)	286 (28.6)		570 (28.5)
A journalist, so they can write a news article about the incident	Definitely able	27 (2.7)	21 (2.1)		25 (2.5)	23 (2.3)		48 (2.4)
	Probably able	52 (5.2)	65 (6.5)		54 (5.4)	63 (6.3)		117 (5.9)
	Not sure	132 (13.2)	142 (14.2)	2.929	133 (13.3)	141 (14.1)	1.418	274 (13.7)
	Probably not able	203 (20.3)	206 (20.6)		202 (20.2)	207 (20.7)		409 (20.4)
	Definitely not able	586 (58.6)	566 (56.6)		586 (58.6)	566 (56.6)		1152 (57.6)
A charity such as the British Red Cross, so they can offer you support	Definitely able	55 (5.5)	62 (6.2)		60 (6.0)	57 (5.7)		117 (5.9)
	Probably able	201 (20.1)	193 (19.3)		183 (18.3)	211 (21.1)		394 (19.7)
	Not sure	281 (28.1)	272 (27.2)	1.593	281 (28.1)	272 (27.2)	3.079	553 (27.7)
	Probably not able	266 (26.6)	285 (28.5)		286 (28.6)	265 (26.5)		551 (27.6)
	Definitely not able	197 (19.7)	188 (18.8)		190 (19.0)	195 (19.5)		385 (19.3)
A health-related Government organisation, so they can send you a questionnaire to find out if you might benefit from extra care or support	Definitely able	95 (9.5)	116 (11.6)		104 (10.4)	107 (10.7)		211 (10.6)
	Probably able	314 (31.4)	393 (39.3)		358 (35.8)	349 (34.9)		707 (35.4)
	Not sure	283 (28.3)	249 (24.9)	21.036***	266 (26.6)	266 (26.6)	0.532	532 (26.6)
	Probably not able	185 (18.5)	147 (14.7)		161 (16.1)	171 (17.1)		332 (16.6)
	Definitely not able	123 (12.3)	95 (9.5)		111 (11.1)	107 (10.7)		218 (10.9)
A specialist NHS team, to provide you with information about ways to get support for any physical or mental health issues	Definitely able	168 (16.8)	205 (20.5)		179 (17.9)	194 (19.4)		373 (18.7)
	Probably able	395 (39.5)	448 (44.8)		429 (42.9)	414 (41.4)		843 (42.1)
	Not sure	218 (21.8)	195 (19.5)	22.844***	209 (20.9)	204 (20.4)	2.120	413 (20.7)
	Probably not able	146 (14.6)	89 (8.9)		111 (11.1)	124 (12.4)		235 (11.8)
	Definitely not able	73 (7.3)	63 (6.3)		72 (7.2)	64 (6.4)		136 (6.8)
A law firm, so they can offer to represent you in a 'no-win, no-fee' claim for compensation	Definitely able	41 (4.1)	33 (3.3)		36 (3.6)	38 (3.8)		74 (3.7)
	Probably able	87 (8.7)	94 (9.4)		91 (9.1)	90 (9.0)		181 (9.1)
	Not sure	164 (16.4)	197 (19.7)	6.087	182 (18.2)	179 (17.9)	0.121	361 (18.1)
	Probably not able	245 (24.5)	253 (25.3)		247 (24.7)	251 (25.1)		498 (24.9)
	Definitely not able	463 (46.3)	423 (42.3)		444 (44.4)	442 (44.2)		886 (44.3)

For peer review only

For peer review only - http://bmjopen.bmj.com/site/about/guidelines.xhtml

Note: ***p<.001

For peer review only

Table 3. Acceptability of police sharing personal data with other agencies, without asking first

Agency requesting personal information and reason for the request		Frequency (%) believing if it is acceptable for the police to share for this reason						
		Shooting	Radiation	χ^2	Pre-incidents	Post-incidents	χ^2	Total
Your GP, so they can check how you are doing	Definitely acceptable	274 (27.4)	324 (32.4)		297 (29.7)	301 (30.1)		598 (29.9)
	Probably acceptable	400 (40.)	404 (40.4)		394 (39.4)	410 (41.0)		804 (40.2)
	Not sure	131 (13.1)	120 (12.0)	10.352*	121 (12.1)	130 (13.0)	3.490	251 (12.6)
	Probably not acceptable	99 (9.9)	82 (8.2)		101 (10.1)	80 (8.0)		181 (9.1)
	Definitely not acceptable	96 (9.6)	70 (7.0)		87 (8.7)	79 (7.9)		166 (8.3)
Your travel insurance company, so they can offer you practical and financial support	Definitely acceptable	107 (10.7)	114 (11.4)		113 (11.3)	108 (10.8)		221 (11.1)
	Probably acceptable	291 (29.1)	271 (27.1)		274 (27.4)	288 (28.8)		562 (28.1)
	Not sure	214 (21.4)	222 (22.2)	2.649	232 (23.2)	204 (20.4)	3.338	436 (21.8)
	Probably not acceptable	175 (17.5)	160 (16.0)		158 (15.8)	177 (17.7)		335 (16.8(
	Definitely not acceptable	213 (21.3)	233 (23.3)		223 (22.3)	223 (22.3)		446 (22.3)
A team of medical researchers from a university, so they can invite you to take part in a study to improve the way future incidents are dealt with	Definitely acceptable	42 (4.2)	70 (7.0)		63 (6.3)	49 (4.9)		112 (5.6)
	Probably acceptable	174 (17.4)	206 (20.6)		203 (20.3)	177 (17.7)		380 (19.0)
	Not sure	221 (22.1)	238 (23.8)	16.001**	214 (21.4)	245 (24.5)	9.797*	459 (22.9)
	Probably not acceptable	268 (26.8)	229 (22.9)		230 (23.0)	267 (26.7)		497 (24.9)
	Definitely not acceptable	295 (29.5)	257 (25.7)		290 (29.0)	262 (26.2)		552 (27.6)
A journalist, so they can write a news article about the incident	Definitely acceptable	17 (1.7)	17 (1.7)		16 (1.6)	18 (1.8)		34 (1.7)
	Probably acceptable	42 (4.2)	49 (4.9)		43 (4.3)	48 (4.8)		91 (4.6)
	Not sure	85 (8.5)	100 (10.0)	2.439	84 (8.4)	101 (10.1)	4.709	185 (9.3)
	Probably not acceptable	143 (14.3)	149 (14.9)		136 (13.6)	156 (15.6)		292 (14.6)
	Definitely not acceptable	713 (71.3)	685 (68.5)		721 (72.1)	677 (67.7)		1398 (69.9)
A charity such as the British Red Cross, so they can offer you support	Definitely acceptable	72 (7.2)	75 (7.5)		75 (7.5)	72 (7.2)		147 (7.4)
	Probably acceptable	277 (27.7)	291 (29.1)		270 (27.0)	298 (29.8)		568 (28.4)
	Not sure	260 (26.0)	245 (24.5)	1.183	254 (25.4)	251 (25.1)	3.131	505 (25.3)
	Probably not acceptable	205 (20.5)	196 (19.6)		199 (19.9)	202 (20.2)		401 (20.1)
	Definitely not acceptable	186 (18.6)	193 (19.3)		202 (20.2)	177 (17.7)		379 (18.9)
A health-related Government organisation, so they can send you a questionnaire to find out if you might benefit from extra care or support	Definitely acceptable	108 (10.8)	152 (15.2)		132 (13.2)	128 (12.8)		260 (13.0)
	Probably acceptable	370 (37.0)	383 (38.3)		361 (36.1)	392 (39.2)		753 (37.6)
	Not sure	229 (22.9)	208 (20.8)	11.036*	225 (22.5)	212 (21.2)	2.502	437 (21.9)
	Probably not acceptable	155 (15.5)	136 (13.6)		153 (15.3)	138 (13.8)		291 (14.6)
	Definitely not acceptable	138 (13.8)	121 (12.1)		129 (12.9)	130 (13.0)		259 (13.0)
A specialist NHS team, to provide you with information about ways to get support for any physical or mental health issues	Definitely acceptable	258 (25.8)	294 (29.4)		271 (27.1)	281 (28.1)		552 (27.6)
	Probably acceptable	438 (43.8)	448 (44.8)		440 (44.0)	446 (44.6)		886 (44.3)
	Not sure	138 (13.8)	126 (12.6)	6.897	139 (13.9)	125 (12.5)	0.978	264 (13.2)
	Probably not acceptable	87 (8.7)	70 (7.0)		79 (7.9)	78 (7.8)		157 (7.9)
	Definitely not acceptable	79 (7.9)	62 (6.2)		71 (7.1)	70 (7.0)		141 (7.1)
A law firm, so they can offer to represent you in a 'no-win, no-fee' claim for compensation	Definitely acceptable	31 (3.1)	28 (2.8)		32 (3.2)	27 (2.7)		59 (3.0)
	Probably acceptable	76 (7.6)	85 (8.5)		83 (8.3)	78 (7.8)		161 (8.1)
	Not sure	137 (13.7)	128 (12.8)	2.796	127 (12.7)	138 (13.8)	6.699	265 (13.3)
	Probably not acceptable	166 (16.6)	189 (18.9)		158 (15.8)	197 (19.7)		355 (17.8)
	Definitely not acceptable	590 (59.0)	570 (57.0)		600 (60.0)	560 (56.0)		1160 (58.0)

Note:*p<.05, **p<.01

For peer review only - http://bmjopen.bmj.com/site/about/guidelines.xhtml

Table 4. Attitudes towards proposed confidential database

If I was caught up in a terrorist incident		Frequency (%) of agreement			
		Pre-incidents	Post-incidents	χ^2	Total
I would want the police to give my contact details to the government organisation so that they can contact me about this service	Strongly agree	209 (20.9)	211 (21.1)		420 (21.0)
	Tend to agree	454 (45.4)	458 (45.8)		912 (45.6)
	Neither	177 (17.7)	175 (17.5)	3.918	352 (17.6)
	Tend to disagree	116 (11.6)	97 (9.7)		213 (10.7)
	Strongly disagree	44 (4.4)	59 (5.9)		103 (5.2)
I would want my name to be included on this database	Strongly agree	143 (14.3)	135 (13.5)		278 (13.9)
	Tend to agree	388 (38.8)	366 (36.6)		754 (37.7)
	Neither	242 (24.2)	265 (26.5)	3.333	507 (25.4)
	Tend to disagree	156 (15.6)	149 (14.9)		305 (15.3)
	Strongly disagree	71 (7.1)	85 (8.5)		156 (7.8)
I would be unhappy if my name was included on this database	Strongly agree	89 (8.9)	102 (10.2)		191 (9.6)
	Tend to agree	181 (18.1)	185 (18.5)		366 (18.3)
	Neither	305 (30.5)	280 (28.0)	2.470	585 (29.3)
	Tend to disagree	323 (32.3)	321 (32.1)		644 (32.2)
	Strongly disagree	102 (10.2)	112 (11.2)		214 (10.7

Note: This data was not split by scenario type, as it regarded a different scenario to that originally posed to participants

For peer review only

Table 5. Attitudes towards contact details being shared with another agency by the police

Imagine the scenario we described occurred and the police decided to share your name and contact information with a health-related government organisation		Frequency (%) of agreement						
		Shooting	Radiation	χ^2	Pre-incidents	Post-incidents	χ^2	Total
I would be concerned that my details would be made public by accident	Strongly agree	290 (29.0)	239 (23.9)		259 (25.9)	270 (27.0)		529 (26.4)
	Tend to agree	360 (26.0)	363 (36.3)		365 (36.5)	358 (35.8)		723 (36.1)
	Neither	201 (20.1)	223 (22.3)	10.004*	217 (21.7)	207 (20.7)	0.742	424 (21.2)
	Tend to disagree	130 (13.0)	143 (14.3)		135 (13.5)	138 (13.8)		273 (13.7)
	Strongly disagree	19 (1.9)	32 (3.2)		24 (2.4)	27 (2.7)		51 (2.6)
I would trust the health organisation to keep my details secure	Strongly agree	152 (15.2)	161 (16.1)		171 (17.1)	142 (14.2)		313 (15.7)
	Tend to agree	363 (36.3)	411 (41.1)		375 (37.5)	399 (39.9)		774 (38.7)
	Neither	266 (26.6)	245 (24.5)	8.537	256 (25.6)	255 (25.5)	7.741	511 (25.6)
	Tend to disagree	164 (16.4)	128 (12.8)		153 (15.3)	139 (13.9)		292 (14.6)
	Strongly disagree	55 (5.5)	55 (5.5)		45 (4.5)	65 (6.5)		110 (5.5)
It would be an invasion of my privacy	Strongly agree	191 (19.1)	159 (15.9)		164 (16.4)	186 (18.6)		350 (17.5)
	Tend to agree	255 (25.5)	227 (22.7)		246 (24.6)	236 (23.6)		482 (24.1)
	Neither	306 (30.6)	288 (28.8)	15.770**	293 (29.3)	301 (30.1)	2.549	594 (29.7)
	Tend to disagree	204 (20.4)	271 (27.1)		244 (24.4)	231 (23.1)		475 (23.8)
	Strongly disagree	44 (4.4)	55 (5.5)		53 (5.3)	46 (4.6)		99 (5.0)
I would be concerned about how my information might be used in the future	Strongly agree	297 (29.7)	251 (25.1)		266 (26.6)	282 (28.2)		548 (27.4)
	Tend to agree	394 (39.4)	414 (41.4)		405 (40.5)	403 (40.3)		808 (40.4)
	Neither	177 (17.7)	203 (20.3)	6.237	192 (19.2)	188 (18.8)	1.755	380 (19.0)
	Tend to disagree	107 (10.7)	109 (10.9)		115 (11.5)	101 (10.1)		216 (10.8)
	Strongly disagree	25 (2.5)	23 (2.3)		22 (2.2)	26 (2.6)		48 (2.4)
I would be concerned that my employer would be told information about me	Strongly agree	116 (11.6)	109 (10.9)		115 (11.5)	110 (11.0)		225 (11.3)
	Tend to agree	232 (23.2)	227 (22.7)		228 (22.2)	231 (23.1)		459 (45.9)
	Neither	281 (28.1)	298 (29.8)	0.847	284 (28.4)	295 (29.5)	0.738	579 (28.9)
	Tend to disagree	261 (26.1)	260 (26.0)		267 (26.7)	254 (25.4)		521 (26.1)
	Strongly disagree	110 (11.0)	106 (10.6)		106 (10.6)	110 (11.0)		216 (10.8)
I would be concerned that my details might be shared by the health organisation with other groups without my permission	Strongly agree	273 (27.3)	233 (23.3)		245 (24.5)	261 (26.1)		506 (25.3)
	Tend to agree	384 (38.4)	380 (38.0)		382 (38.2)	382 (38.2)		764 (38.2)
	Neither	201 (20.1)	245 (24.5)	8.629	227 (22.7)	219 (21.9)	2.127	446 (22.3)
	Tend to disagree	111 (11.1)	118 (11.8)		114 (11.4)	115 (11.5)		229 (11.5)
	Strongly disagree	31 (3.1)	24 (2.4)		32 (3.2)	23 (2.3)		55 (2.8)
I would want to be kept informed about how my information was being used	Strongly agree	509 (50.9)	489 (48.9)		518 (51.8)	480 (48.0)		998 (49.9)
	Tend to agree	340 (34.0)	357 (35.7)		339 (33.9)	358 (35.8)		697 (34.9)
	Neither	114 (11.4)	128 (12.8)	9.692*	109 (10.9)	133 (13.3)	5.162	242 (12.1)
	Tend to disagree	24 (2.4)	24 (2.4)		27 (2.7)	21 (2.1)		48 (2.4)
	Strongly disagree	13 (1.3)	2 (0.2)		7 (0.7)	8 (0.8)		15 (0.8)
It wouldn't bother me at all	Strongly agree	58 (5.8)	72 (7.2)		64 (6.4)	66 (6.6)		130 (6.5)
	Tend to agree	204 (20.4)	222 (22.2)		202 (20.3)	224 (22.4)		426 (21.3)
	Neither	308 (30.8)	330 (33.0)	7.131	332 (33.2)	306 (30.6)	2.232	638 (31.9)
	Tend to disagree	270 (27.0)	245 (24.5)		257 (25.7)	258 (25.8)		515 (25.8)
	Strongly disagree	160 (16.0)	131 (13.1)		145 (14.5)	146 (14.6)		291 (14.6)
I would be reassured that a health organisation was looking out for me	Strongly agree	107 (10.7)	148 (14.8)		137 (13.7)	118 (11.8)		255 (12.8)
	Tend to agree	425 (42.5)	427 (42.7)		415 (41.5)	437 (43.7)		852 (42.6)
	Neither	305 (30.5)	302 (30.2)	12.829*	319 (31.9)	288 (28.8)	7.791	607 (60.7)
	Tend to disagree	112 (11.2)	79 (7.9)		91 (9.1)	100 (10.0)		191 (9.6)
	Strongly disagree	51 (5.1)	44 (4.4)		38 (3.8)	57 (5.7)		95 (4.8)
I would be concerned that my GP might be told	Strongly agree	44 (4.4)	42 (4.2)		41 (4.1)	45 (4.5)		86 (4.3)
	Tend to agree	102 (10.2)	89 (8.9)		90 (9.0)	101 (10.1)		191 (9.6)
	Neither	296 (29.6)	277 (27.7)	2.679	292 (29.2)	281 (28.1)	3.554	573 (28.7)
	Tend to disagree	369 (36.9)	397 (39.7)		397 (39.7)	369 (36.9)		766 (38.3)
	Strongly disagree	189 (18.9)	195 (19.5)		180 (18.0)	204 (20.4)		384 (19.2)

For peer review only

Note: *p<.05, **p<.01

For peer review only

For peer review only - http://bmjopen.bmj.com/site/about/guidelines.xhtml

Supplementary material 1: KCL Disaster Data Protection Topline Results for Wave 1Supplementary material 1 - KCL Disaster Data Protection Topline Results for Wave 1

KCL Disaster Data Protection Survey
Topline results

- Results are based on interviews with 1000 members of the public aged 18-65 across England. Interviews were conducted between 9 March 2017 and 15 March 2017.
- All interviews were conducted via Ipsos MORI's online panel.
- The survey data is unweighted but based on quotas on age, gender, region and working status to reflect the profile of adults aged 18-65 in England, with up to 10% flexibility. Weights are available in the SPSS file for the whole sample, Sample A and Sample B.
- Results are based on all respondents (1000) unless otherwise stated. Bases specified are unweighted.
- Where percentages do not sum to 100, this may be due to respondents being able to select multiple responses, computer rounding or the exclusion of 'don't know'/ not stated.
- An asterisk (*) represents a value of less than half or one percent, but greater than zero.
- The response rate for this survey was 10% (1000 responses from 10004 people that the link was emailed to).
- 25 participants ended the survey without completing it (and were not included in the data); 26 people were excluded from the data due to being identified as 'speeding' or straightlining'; i.e. completing the survey too quickly to have given genuine, considered answers, or providing identical answers to five or more consecutive questions where this was possible.

Q1. Please select your date of birth (recoded into age categories):

	%
18-24	12
25-34	23
35-44	21
45-54	23
55-65	21

Q2. Please state the gender you identify yourself with:

	%
Male	49
Female	51

Q3. In which of the following regions do you live?

	%
North East	5
North West	14
Yorkshire and Humberside	10
West Midlands	10
East Midlands	9
East of England	9
South West	10
South East	17
London	18

For peer review only - http://bmjopen.bmj.com/site/about/guidelines.xhtml

Supplementary material 1: KCL Disaster Data Protection Topline Results for Wave 1Supplementary material 1 - KCL Disaster Data Protection Topline Results for Wave 1

Q4. Which of the following best describes your employment status?

	%
Working – full time (30 or more hours a week)	50
Working – part-time (less than 30 hours a week)	15
Self-employed	7
Unemployed – looking for a job	5
Unemployed – not looking for a job/long-term sick or disabled/Housewife/husband/Full-time carer	11
Retired	8
Pupil/Student/In full-time education	5
**NET: Working**	72
**NET: Not working**	28

We are interested in people's views about sharing information following a major incident such as a terrorist attack. The researchers have no reason to think that an attack is going to happen. This research is one of a number of studies that academics at King's College London are conducting on how people might react to a range of different issues.

SAMPLE A:
Imagine that you are on holiday in another country and witness a terrorist shooting. Some people are badly injured but you are not harmed and neither is anyone you know well. The British government arranges for you to be flown home, along with other British nationals who were in the area. When you arrive back in Britain, a police officer at the airport records your name, address, phone number and email address. The following questions ask about things that might happen next.

SAMPLE B:
Imagine that you are on holiday in another country and a place you visit is discovered by police to be contaminated by radioactive material. The police believe this is linked to a terrorist group. Some people are badly injured but you are not harmed and neither is anyone you know well. The British government arranges for you to be flown home, along with other British nationals who were in the area. When you arrive back in Britain, a police officer at the airport records your name, address, phone number and email address. The following questions ask about things that might happen next.

Q5. (TOTAL) Following an incident like this, various groups or individuals might ask the police to tell them who was caught up in the incident. Please say whether you think that the police are or are not currently able to share your name and contact details with these groups or people following an incident like this, without checking with you first.

1. **Your GP, so they can check how you are doing**

	% (TOTAL – Base = 1000)	% (SAMPLE A – Base = 500)	% (SAMPLE B – Base = 500)
Definitely able to share	18	14	22
Probably able to share	36	37	35
Not sure	21	21	20
Probably not able to share	15	18	13
Definitely not able to share	10	10	10

2. **Your travel insurance company, so they can offer you practical and financial support**

	% (TOTAL – Base = 1000)	% (SAMPLE A – Base = 500)	% (SAMPLE B – Base = 500)
Definitely able to share	8	8	9
Probably able to share	24	25	23
Not sure	27	27	28
Probably not able to share	24	26	22

For peer review only - http://bmjopen.bmj.com/site/about/guidelines.xhtml

Supplementary material 1: KCL Disaster Data Protection Topline Results for Wave 1Supplementary material 1 - KCL Disaster Data Protection Topline Results for Wave 1

Definitely not able to share	17	16	19

3. A team of medical researchers from a university, so they can invite you to take part in a study to improve the way future incidents are dealt with

	% (TOTAL – Base = 1000)	% (SAMPLE A – Base = 500)	% (SAMPLE B – Base = 500)
Definitely able to share	5	4	6
Probably able to share	14	14	13
Not sure	22	22	23
Probably not able to share	31	31	30
Definitely not able to share	28	29	28

4. A journalist, so they can write a news article about the incident

	% (TOTAL – Base = 1000)	% (SAMPLE A – Base = 500)	% (SAMPLE B – Base = 500)
Definitely able to share	3	3	2
Probably able to share	5	4	7
Not sure	13	13	13
Probably not able to share	20	22	19
Definitely not able to share	59	58	59

5. A charity, such as the British Red Cross, so they can offer you support

	% (TOTAL – Base = 1000)	% (SAMPLE A – Base = 500)	% (SAMPLE B – Base = 500)
Definitely able to share	6	5	7
Probably able to share	18	20	16
Not sure	28	29	28
Probably not able to share	29	28	30
Definitely not able to share	19	18	20

6. A health-related Government organisation, so they can send you a questionnaire to find out if you might benefit from extra care or support.

	% (TOTAL – Base = 1000)	% (SAMPLE A – Base = 500)	% (SAMPLE B – Base = 500)
Definitely able to share	10	8	13
Probably able to share	36	33	39
Not sure	27	29	25
Probably not able to share	16	18	15
Definitely not able to share	11	13	9

7. A specialist NHS team, to provide you with information about ways to get support for any physical or mental health issues.

	% (TOTAL – Base = 1000)	% (SAMPLE A – Base = 500)	% (SAMPLE B – Base = 500)
Definitely able to share	18	14	22
Probably able to share	43	41	45
Not sure	21	25	17
Probably not able to share	11	14	9
Definitely not able to share	7	7	7

For peer review only - http://bmjopen.bmj.com/site/about/guidelines.xhtml

Supplementary material 1: KCL Disaster Data Protection Topline Results for Wave 1Supplementary material 1 - KCL Disaster Data Protection Topline Results for Wave 1

8. A law firm, so they can offer to represent you in a 'no-win, no-fee' claim for compensation

	% (TOTAL – Base = 1000)	% (SAMPLE A – Base = 500)	% (SAMPLE B – Base = 500)
Definitely able to share	4	3	4
Probably able to share	9	9	9
Not sure	18	17	20
Probably not able to share	25	23	27
Definitely not able to share	44	48	41

Q6. Following an incident like this, various groups or individuals might ask the police to tell them who was caught up in the incident. For each of the following groups, please say whether you think that is acceptable or not acceptable for the police to share your name and contact details with these groups or people following an incident like this, without checking with you first;

1. Your GP, so they can check how you are doing

	% (TOTAL – Base = 1000)	% (SAMPLE A – Base = 500)	% (SAMPLE B – Base = 500)
Definitely acceptable to share	30	26	33
Probably acceptable to share	39	38	40
Not sure	12	14	10
Probably not acceptable to share	10	11	9
Definitely not acceptable to share	9	10	8

2. Your travel insurance company, so they can offer you practical and financial support

	% (TOTAL – Base = 1000)	% (SAMPLE A – Base = 500)	% (SAMPLE B – Base = 500)
Definitely acceptable to share	11	9	13
Probably acceptable to share	27	30	25
Not sure	23	23	24
Probably not acceptable to share	16	16	16
Definitely not acceptable to share	22	22	22

3. A team of medical researchers from a university, so they can invite you to take part in a study to improve the way future incidents are dealt with

	% (TOTAL – Base = 1000)	% (SAMPLE A – Base = 500)	% (SAMPLE B – Base = 500)
Definitely acceptable to share	6	5	8
Probably acceptable to share	20	19	22
Not sure	21	22	21
Probably not acceptable to share	23	23	23
Definitely not acceptable to share	29	31	27

4. A journalist, so they can write a news article about the incident

	% (TOTAL – Base = 1000)	% (SAMPLE A – Base = 500)	% (SAMPLE B – Base = 500)
Definitely acceptable to share	2	1	2
Probably acceptable to share	4	4	4
Not sure	8	10	7
Probably not acceptable to share	14	13	14
Definitely not acceptable to share	72	72	73

For peer review only - http://bmjopen.bmj.com/site/about/guidelines.xhtml

Supplementary material 1: KCL Disaster Data Protection Topline Results for Wave 1Supplementary material 1 - KCL Disaster Data Protection Topline Results for Wave 1

5. A charity, such as the British Red Cross, so they can offer you support

	% (TOTAL – Base = 1000)	% (SAMPLE A – Base = 500)	% (SAMPLE B – Base = 500)
Definitely acceptable to share	8	7	8
Probably acceptable to share	27	27	27
Not sure	25	27	24
Probably not acceptable to share	20	19	20
Definitely not acceptable to share	20	20	20

6. A health-related Government organisation, so they can send you a questionnaire to find out if you might benefit from extra care or support.

	% (TOTAL – Base = 1000)	% (SAMPLE A – Base = 500)	% (SAMPLE B – Base = 500)
Definitely acceptable to share	13	10	17
Probably acceptable to share	36	35	37
Not sure	23	25	20
Probably not acceptable to share	15	16	15
Definitely not acceptable to share	13	14	12

7. A specialist NHS team, to provide you with information about ways to get support for any physical or mental health issues.

	% (TOTAL – Base = 1000)	% (SAMPLE A – Base = 500)	% (SAMPLE B – Base = 500)
Definitely acceptable to share	27	23	31
Probably acceptable to share	44	43	45
Not sure	14	16	12
Probably not acceptable to share	8	9	7
Definitely not acceptable to share	7	8	6

8. A law firm, so they can offer to represent you in a 'no-win, no-fee' claim for compensation

	% (TOTAL – Base = 1000)	% (SAMPLE A – Base = 500)	% (SAMPLE B – Base = 500)
Definitely acceptable to share	3	3	4
Probably acceptable to share	8	8	9
Not sure	13	14	11
Probably not acceptable to share	16	14	18
Definitely not acceptable to share	60	61	59

Following a terrorist incident, people sometimes develop mental health problems as a result of their experiences. To provide help, the Government sometimes sets up a specialist NHS service to offer support to those who need it. A government organisation would be asked to help with this by putting together a confidential database of people who were present and then writing to them with information about the NHS service and a short mental health questionnaire.

Q7. To what extent do you agree or disagree with the following statement. If I was caught up in a terrorist incident, I would want the police to give my contact details to the government organisation so that they can contact me about this service.

	%
Strongly agree	21
Tend to agree	45
Neither agree nor disagree	18
Tend to disagree	12
Strongly disagree	4

For peer review only - http://bmjopen.bmj.com/site/about/guidelines.xhtml

Supplementary material 1: KCL Disaster Data Protection Topline Results for Wave 1Supplementary material 1 - KCL Disaster Data Protection Topline Results for Wave 1

Q8. And why do you say that? OPEN-ENDED RESPONSES

Q9. To what extent do you agree or disagree with the following statement: If I was caught up in a terrorist incident, I would want my name to be included on this database.

	%
Strongly agree	14
Tend to agree	39
Neither agree nor disagree	24
Tend to disagree	16
Strongly disagree	7

Q10. To what extent do you agree or disagree with the following statement: If I was caught up in a terrorist incident, I would be unhappy if my name was included on this database.

	%
Strongly agree	9
Tend to agree	18
Neither agree nor disagree	31
Tend to disagree	32
Strongly disagree	10

Q11A. Imagine the scenario we described occurred and the police decided to share your name and contact information with a health-related government organisation. Please say how much you agree or disagree with the following statements:

1. **I would be concerned that my details would be made public by accident**

	% (TOTAL – Base = 1000)	% (SAMPLE A – Base = 500)	% (SAMPLE B – Base = 500)
Strongly agree	26	28	24
Tend to agree	37	38	35
Neither agree nor disagree	22	20	23
Tend to disagree	14	13	14
Strongly disagree	2	2	3

2. **I would trust the health organisation to keep my details secure**

	% (TOTAL – Base = 1000)	% (SAMPLE A – Base = 500)	% (SAMPLE B – Base = 500)
Strongly agree	17	16	19
Tend to agree	38	36	39
Neither agree nor disagree	26	27	25
Tend to disagree	15	18	13
Strongly disagree	5	4	5

3. **It would be an invasion of my privacy**

	% (TOTAL – Base = 1000)	% (SAMPLE A – Base = 500)	% (SAMPLE B – Base = 500)
Strongly agree	16	17	15
Tend to agree	25	26	23
Neither agree nor disagree	29	32	27
Tend to disagree	24	21	28
Strongly disagree	5	4	6

For peer review only - http://bmjopen.bmj.com/site/about/guidelines.xhtml

Supplementary material 1: KCL Disaster Data Protection Topline Results for Wave 1Supplementary material 1 - KCL Disaster Data Protection Topline Results for Wave 1

4. I would be concerned about how my information might be used in the future

	% (TOTAL – Base = 1000)	% (SAMPLE A – Base = 500)	% (SAMPLE B – Base = 500)
Strongly agree	27	30	24
Tend to agree	41	41	40
Neither agree nor disagree	19	16	22
Tend to disagree	12	11	12
Strongly disagree	2	2	2

5. I would be concerned that my employer would be told information about me

	% (TOTAL – Base = 1000)	% (SAMPLE A – Base = 500)	% (SAMPLE B – Base = 500)
Strongly agree	12	12	11
Tend to agree	23	25	21
Neither agree nor disagree	28	28	29
Tend to disagree	27	25	29
Strongly disagree	11	11	11

Q11B. Imagine the scenario we described occurred and the police decided to share your name and contact information with a health-related government organisation. Please say how much you agree or disagree with the following statements:

1. I would be concerned that my details might be shared by the health organisation with other groups without my permission

	% (TOTAL – Base = 1000)	% (SAMPLE A – Base = 500)	% (SAMPLE B – Base = 500)
Strongly agree	25	27	22
Tend to agree	38	36	41
Neither agree nor disagree	23	22	23
Tend to disagree	11	11	12
Strongly disagree	3	4	3

2. I would want to be kept informed about how my information was being used

	% (TOTAL – Base = 1000)	% (SAMPLE A – Base = 500)	% (SAMPLE B – Base = 500)
Strongly agree	52	52	51
Tend to agree	34	32	35
Neither agree nor disagree	11	11	11
Tend to disagree	3	3	2
Strongly disagree	1	1	*

3. It wouldn't bother me at all

	% (TOTAL – Base = 1000)	% (SAMPLE A – Base = 500)	% (SAMPLE B – Base = 500)
Strongly agree	6	5	7
Tend to agree	20	19	21
Neither agree nor disagree	33	32	35
Tend to disagree	26	28	24
Strongly disagree	15	16	13

For peer review only - http://bmjopen.bmj.com/site/about/guidelines.xhtml

For peer review only

Supplementary material 1: KCL Disaster Data Protection Topline Results for Wave 1Supplementary material 1 - KCL Disaster Data Protection Topline Results for Wave 1

4. I would be reassured that a health organisation was looking out for me

	% (TOTAL – Base = 1000)	% (SAMPLE A – Base = 500)	% (SAMPLE B – Base = 500)
Strongly agree	14	11	17
Tend to agree	42	43	40
Neither agree nor disagree	32	32	31
Tend to disagree	9	10	8
Strongly disagree	4	4	4

5. I would be concerned that my GP might be told

	% (TOTAL – Base = 1000)	% (SAMPLE A – Base = 500)	% (SAMPLE B – Base = 500)
Strongly agree	4	4	4
Tend to agree	9	11	7
Neither agree nor disagree	29	30	28
Tend to disagree	40	38	41
Strongly disagree	18	17	19

Q12. Please state the highest level of education you have achieved:

	%
Left school without qualifications	3
Secondary education (O-level/GCSE/A-level)	42
Higher education (BSc, BA/higher qualification)	54
Prefer not to say	1

For peer review only

For peer review only - http://bmjopen.bmj.com/site/about/guidelines.xhtml

Supplementary material 2 - KCL Disaster Data Protection Topline Results for Wave 2

KCL Disaster Data Protection Survey – Wave 2
Topline results

- Results are based on interviews with 1000 members of the public aged 18-65 across England. Interviews were conducted between 3 and 10 July 2017.
- All interviews were conducted via Ipsos MORI's online panel.
- The survey data is unweighted but based on quotas on age, gender, region and working status (with age and gender interlocked) to reflect the profile of adults aged 18-65 in England, with up to 10% flexibility on region and working status. Weights are available in the SPSS file for the whole sample, Sample A and Sample B, for both the first and second wave and for both waves combined.
- Results are based on all respondents (1000) unless otherwise stated. Bases specified are unweighted.
- Where percentages do not sum to 100, this may be due to respondents being able to select multiple responses, computer rounding or the exclusion of 'don't know'/ not stated.
- An asterisk (*) represents a value of less than half of one percent, but greater than zero.
- 14,735 people were invited to take part in the survey.
- 30 participants ended the survey without completing it (and were not included in the data); 19 people were excluded from the data due to being identified as 'speeding' or straightlining'; i.e. completing the survey too quickly to have given genuine, considered answers, or providing identical answers to five or more consecutive questions where this was possible.
- This work has been carried out in accordance with the requirements of the international quality standard for Market Research, ISO 20252:2012, and with the standard Ipsos MORI Terms and Conditions which can be found at http://www.ipsos-mori.com/terms. © Ipsos MORI 2017

Q1. Please select your date of birth (recoded into age categories):

	%
18-24	14.4
25-34	22.2
35-44	20.7
45-54	22.6
55-65	20.1

Q2. Please state the gender you identify yourself with:

	%
Male	49.6
Female	50.4

Q3. In which of the following regions do you live?

	%
North East	4.6
North West	13.5
Yorkshire and Humberside	10.1
West Midlands	10.1
East Midlands	8.4
East of England	9.9
South West	9.0
South East	16.6

For peer review only - http://bmjopen.bmj.com/site/about/guidelines.xhtml

London	17.8

Q4. Which of the following best describes your employment status?

	%
Working – full time (30 or more hours a week)	50.9
Working – part-time (less than 30 hours a week)	14.0
Self-employed	7.8
Unemployed – looking for a job	5.2
Unemployed – not looking for a job/long-term sick or disabled/Housewife/husband/Full-time carer	10.3
Retired	5.6
Pupil/Student/In full-time education	6.2
**NET: Working**	72.7
**NET: Not working**	27.3

We are interested in people's views about sharing information following a major incident such as a terrorist attack. The researchers have no reason to think that an attack is going to happen. This research is one of a number of studies that academics at King's College London are conducting on how people might react to a range of different issues.

SAMPLE A:
Imagine that you are on holiday in another country and witness a terrorist shooting. Some people are badly injured but you are not harmed and neither is anyone you know well. The British government arranges for you to be flown home, along with other British nationals who were in the area. When you arrive back in Britain, a police officer at the airport records your name, address, phone number and email address. The following questions ask about things that might happen next.

SAMPLE B:
Imagine that you are on holiday in another country and a place you visit is discovered by police to be contaminated by radioactive material. The police believe this is linked to a terrorist group. Some people are badly injured but you are not harmed and neither is anyone you know well. The British government arranges for you to be flown home, along with other British nationals who were in the area. When you arrive back in Britain, a police officer at the airport records your name, address, phone number and email address. The following questions ask about things that might happen next.

Q5. Following an incident like this, various groups or individuals might ask the police to tell them who was caught up in the incident. Please say whether you think that the police are or are not currently able to share your name and contact details with these groups or people following an incident like this, without checking with you first.

1. **Your GP, so they can check how you are doing**

	% (TOTAL – Base = 1000)	% (SAMPLE A – Base = 500)	% (SAMPLE B – Base = 500)
Definitely able to share	19.6	19.8	19.4
Probably able to share	34.1	28.8	39.4
Not sure	20.9	21.2	20.6
Probably not able to share	15.2	18.8	11.6
Definitely not able to share	10.2	11.4	9.0

2. **Your travel insurance company, so they can offer you practical and financial support**

	% (TOTAL – Base = 1000)	% (SAMPLE A – Base = 500)	% (SAMPLE B – Base = 500)
Definitely able to share	10.6	11.8	9.4
Probably able to share	22.9	22.4	23.4
Not sure	24.6	24.2	25.0
Probably not able to share	23.2	22.8	23.6

For peer review only - http://bmjopen.bmj.com/site/about/guidelines.xhtml

Supplementary material 2 - KCL Disaster Data Protection Topline Results for Wave 2

Definitely not able to share	18.7	18.8	18.6

3. A team of medical researchers from a university, so they can invite you to take part in a study to improve the way future incidents are dealt with

	% (TOTAL – Base = 1000)	% (SAMPLE A – Base = 500)	% (SAMPLE B – Base = 500)
Definitely able to share	4.4	3.8	5.0
Probably able to share	13.2	10.6	15.8
Not sure	23.7	23.6	23.8
Probably not able to share	30.1	31.4	28.8
Definitely not able to share	28.6	30.6	26.6

4. A journalist, so they can write a news article about the incident

	% (TOTAL – Base = 1000)	% (SAMPLE A – Base = 500)	% (SAMPLE B – Base = 500)
Definitely able to share	2.3	2.4	2.2
Probably able to share	6.3	6.2	6.4
Not sure	14.1	13.2	15.0
Probably not able to share	20.7	19.0	22.4
Definitely not able to share	56.6	59.2	54.0

5. A charity, such as the British Red Cross, so they can offer you support

	% (TOTAL – Base = 1000)	% (SAMPLE A – Base = 500)	% (SAMPLE B – Base = 500)
Definitely able to share	5.7	5.6	5.8
Probably able to share	21.1	20.0	22.2
Not sure	27.2	27.6	26.8
Probably not able to share	26.5	25.6	27.4
Definitely not able to share	19.5	21.2	17.8

6. A health-related Government organisation, so they can send you a questionnaire to find out if you might benefit from extra care or support.

	% (TOTAL – Base = 1000)	% (SAMPLE A – Base = 500)	% (SAMPLE B – Base = 500)
Definitely able to share	10.7	11.0	10.4
Probably able to share	34.9	29.8	40.0
Not sure	26.6	28.0	25.2
Probably not able to share	17.1	19.4	14.8
Definitely not able to share	10.7	11.8	9.6

7. A specialist NHS team, to provide you with information about ways to get support for any physical or mental health issues.

	% (TOTAL – Base = 1000)	% (SAMPLE A – Base = 500)	% (SAMPLE B – Base = 500)
Definitely able to share	19.4	19.8	19.0
Probably able to share	41.4	38.0	44.8
Not sure	20.4	19.0	21.8
Probably not able to share	12.4	15.6	9.2
Definitely not able to share	6.4	7.6	5.2

For peer review only

Supplementary material 2 - KCL Disaster Data Protection Topline Results for Wave 2

8. A law firm, so they can offer to represent you in a 'no-win, no-fee' claim for compensation

	% (TOTAL – Base = 1000)	% (SAMPLE A – Base = 500)	% (SAMPLE B – Base = 500)
Definitely able to share	3.8	5.0	2.6
Probably able to share	9.0	8.4	9.6
Not sure	17.9	16.0	19.8
Probably not able to share	25.1	26.2	24.0
Definitely not able to share	44.2	44.4	44.0

Q6. Following an incident like this, various groups or individuals might ask the police to tell them who was caught up in the incident. For each of the following groups, please say whether you think that is acceptable or not acceptable for the police to share your name and contact details with these groups or people following an incident like this, without checking with you first;

1. Your GP, so they can check how you are doing

	% (TOTAL – Base = 1000)	% (SAMPLE A – Base = 500)	% (SAMPLE B – Base = 500)
Definitely acceptable to share	30.1	28.6	31.6
Probably acceptable to share	41.0	41.6	40.4
Not sure	13.0	12.0	14.0
Probably not acceptable to share	8.0	8.4	7.6
Definitely not acceptable to share	7.9	9.4	6.4

2. Your travel insurance company, so they can offer you practical and financial support

	% (TOTAL – Base = 1000)	% (SAMPLE A – Base = 500)	% (SAMPLE B – Base = 500)
Definitely acceptable to share	10.8	12.2	9.4
Probably acceptable to share	28.8	28.0	29.6
Not sure	20.4	20.0	20.8
Probably not acceptable to share	17.7	19.4	16.0
Definitely not acceptable to share	22.3	20.4	24.2

3. A team of medical researchers from a university, so they can invite you to take part in a study to improve the way future incidents are dealt with

	% (TOTAL – Base = 1000)	% (SAMPLE A – Base = 500)	% (SAMPLE B – Base = 500)
Definitely acceptable to share	4.9	3.4	6.4
Probably acceptable to share	17.7	16.2	19.2
Not sure	24.5	22.4	26.6
Probably not acceptable to share	26.7	30.2	23.2
Definitely not acceptable to share	26.2	27.8	24.6

4. A journalist, so they can write a news article about the incident

	% (TOTAL – Base = 1000)	% (SAMPLE A – Base = 500)	% (SAMPLE B – Base = 500)
Definitely acceptable to share	1.8	2.0	1.6
Probably acceptable to share	4.8	4.2	5.4
Not sure	10.1	7.4	12.8
Probably not acceptable to share	15.6	15.4	15.8
Definitely not acceptable to share	67.7	71.0	64.4

For peer review only - http://bmjopen.bmj.com/site/about/guidelines.xhtml

Supplementary material 2 - KCL Disaster Data Protection Topline Results for Wave 2

5. **A charity, such as the British Red Cross, so they can offer you support**

	% (TOTAL – Base = 1000)	% (SAMPLE A – Base = 500)	% (SAMPLE B – Base = 500)
Definitely acceptable to share	7.2	7.0	7.4
Probably acceptable to share	29.8	28.8	30.8
Not sure	25.1	25.4	24.8
Probably not acceptable to share	20.2	21.6	18.8
Definitely not acceptable to share	17.7	17.2	18.2

6. **A health-related Government organisation, so they can send you a questionnaire to find out if you might benefit from extra care or support.**

	% (TOTAL – Base = 1000)	% (SAMPLE A – Base = 500)	% (SAMPLE B – Base = 500)
Definitely acceptable to share	12.8	11.8	13.8
Probably acceptable to share	39.2	39.0	39.4
Not sure	21.2	20.6	21.8
Probably not acceptable to share	13.8	15.2	12.4
Definitely not acceptable to share	13.0	13.4	12.6

7. **A specialist NHS team, to provide you with information about ways to get support for any physical or mental health issues.**

	% (TOTAL – Base = 1000)	% (SAMPLE A – Base = 500)	% (SAMPLE B – Base = 500)
Definitely acceptable to share	28.1	28.2	28.0
Probably acceptable to share	44.6	44.6	44.6
Not sure	12.5	11.4	13.6
Probably not acceptable to share	7.8	8.2	7.4
Definitely not acceptable to share	7.0	7.6	6.4

8. **A law firm, so they can offer to represent you in a 'no-win, no-fee' claim for compensation**

	% (TOTAL – Base = 1000)	% (SAMPLE A – Base = 500)	% (SAMPLE B – Base = 500)
Definitely acceptable to share	2.7	3.6	1.8
Probably acceptable to share	7.8	7.2	8.4
Not sure	13.8	13.2	14.4
Probably not acceptable to share	19.7	19.4	20.0
Definitely not acceptable to share	56.0	56.6	55.4

Following a terrorist incident, people sometimes develop mental health problems as a result of their experiences. To provide help, the Government sometimes sets up a specialist NHS service to offer support to those who need it. A government organisation would be asked to help with this by putting together a confidential database of people who were present and then writing to them with information about the NHS service and a short mental health questionnaire.

Q7. To what extent do you agree or disagree with the following statement. If I was caught up in a terrorist incident, I would want the police to give my contact details to the government organisation so that they can contact me about this service.

	%
Strongly agree	21.1
Tend to agree	45.8
Neither agree nor disagree	17.5
Tend to disagree	9.7
Strongly disagree	5.9

For peer review only - http://bmjopen.bmj.com/site/about/guidelines.xhtml

Supplementary material 2 - KCL Disaster Data Protection Topline Results for Wave 2

Q8. And why do you say that? OPEN-ENDED RESPONSES

Q9. To what extent do you agree or disagree with the following statement: If I was caught up in a terrorist incident, I would want my name to be included on this database.

	%
Strongly agree	13.5
Tend to agree	36.6
Neither agree nor disagree	26.5
Tend to disagree	14.9
Strongly disagree	8.5

Q10. To what extent do you agree or disagree with the following statement: If I was caught up in a terrorist incident, I would be unhappy if my name was included on this database.

	%
Strongly agree	10.2
Tend to agree	18.5
Neither agree nor disagree	28.0
Tend to disagree	32.1
Strongly disagree	11.2

Q11A. Imagine the scenario we described occurred and the police decided to share your name and contact information with a health-related government organisation. Please say how much you agree or disagree with the following statements:

1. I would be concerned that my details would be made public by accident

	% (TOTAL – Base = 1000)	% (SAMPLE A – Base = 500)	% (SAMPLE B – Base = 500)
Strongly agree	27.0	30.2	23.8
Tend to agree	35.8	34.4	37.2
Neither agree nor disagree	20.7	19.8	21.6
Tend to disagree	13.8	13.4	14.2
Strongly disagree	2.7	2.2	3.2

2. I would trust the health organisation to keep my details secure

	% (TOTAL – Base = 1000)	% (SAMPLE A – Base = 500)	% (SAMPLE B – Base = 500)
Strongly agree	14.2	14.8	13.6
Tend to agree	39.9	36.8	43.0
Neither agree nor disagree	25.5	26.6	24.4
Tend to disagree	13.9	15.2	12.6
Strongly disagree	6.5	6.6	6.4

3. It would be an invasion of my privacy

	% (TOTAL – Base = 1000)	% (SAMPLE A – Base = 500)	% (SAMPLE B – Base = 500)
Strongly agree	18.6	20.8	16.4
Tend to agree	23.6	25.2	22.0
Neither agree nor disagree	30.1	29.4	30.8
Tend to disagree	23.1	20.2	26.0
Strongly disagree	4.6	4.4	4.8

For peer review only

4. **I would be concerned about how my information might be used in the future**

	% (TOTAL – Base = 1000)	% (SAMPLE A – Base = 500)	% (SAMPLE B – Base = 500)
Strongly agree	28.2	29.8	26.6
Tend to agree	40.3	38.2	42.4
Neither agree nor disagree	18.8	19.0	18.6
Tend to disagree	10.1	10.4	9.8
Strongly disagree	2.6	2.6	2.6

5. **I would be concerned that my employer would be told information about me**

	% (TOTAL – Base = 1000)	% (SAMPLE A – Base = 500)	% (SAMPLE B – Base = 500)
Strongly agree	11.0	11.6	10.4
Tend to agree	23.1	21.4	24.8
Neither agree nor disagree	29.5	28.2	30.8
Tend to disagree	25.4	27.4	23.4
Strongly disagree	11.0	11.4	10.6

Q11B. Imagine the scenario we described occurred and the police decided to share your name and contact information with a health-related government organisation. Please say how much you agree or disagree with the following statements:

1. **I would be concerned that my details might be shared by the health organisation with other groups without my permission**

	% (TOTAL – Base = 1000)	% (SAMPLE A – Base = 500)	% (SAMPLE B – Base = 500)
Strongly agree	26.1	27.2	25.0
Tend to agree	38.2	41.0	35.4
Neither agree nor disagree	21.9	18.2	25.6
Tend to disagree	11.5	11.0	12.0
Strongly disagree	2.3	2.6	2.0

2. **I would want to be kept informed about how my information was being used**

	% (TOTAL – Base = 1000)	% (SAMPLE A – Base = 500)	% (SAMPLE B – Base = 500)
Strongly agree	48.0	49.6	46.4
Tend to agree	35.8	35.6	36.0
Neither agree nor disagree	13.3	12.0	14.6
Tend to disagree	2.1	1.4	2.8
Strongly disagree	0.8	1.4	*

3. **It wouldn't bother me at all**

	% (TOTAL – Base = 1000)	% (SAMPLE A – Base = 500)	% (SAMPLE B – Base = 500)
Strongly agree	6.6	6.2	7.0
Tend to agree	22.4	21.8	23.0
Neither agree nor disagree	30.6	29.8	31.4
Tend to disagree	25.8	26.4	25.2
Strongly disagree	14.6	15.8	13.4

For peer review only - http://bmjopen.bmj.com/site/about/guidelines.xhtml

Supplementary material 2 - KCL Disaster Data Protection Topline Results for Wave 2

4. I would be reassured that a health organisation was looking out for me

	% (TOTAL – Base = 1000)	% (SAMPLE A – Base = 500)	% (SAMPLE B – Base = 500)
Strongly agree	11.8	10.8	12.8
Tend to agree	43.7	42.0	45.4
Neither agree nor disagree	28.8	28.6	29.0
Tend to disagree	10.0	12.2	7.8
Strongly disagree	5.7	6.4	5.0

5. I would be concerned that my GP might be told

	% (TOTAL – Base = 1000)	% (SAMPLE A – Base = 500)	% (SAMPLE B – Base = 500)
Strongly agree	4.5	4.4	4.6
Tend to agree	10.1	9.6	10.6
Neither agree nor disagree	28.1	29.2	27.0
Tend to disagree	36.9	35.6	38.2
Strongly disagree	20.4	21.2	19.6

Q12. Please state the highest level of education you have achieved:

	%
Left school without qualifications	2.7
Secondary education (O-level/GCSE/A-level)	41.3
Higher education (BSc, BA/higher qualification)	55.5
Prefer not to say	0.5

For peer review only

STROBE Statement—Checklist of items that should be included in reports of ***cross-sectional studies***

	Item No	Recommendation	Page No(s)
**Title and abstract**	1	(*a*) Indicate the study's design with a commonly used term in the title or the abstract	1
		(*b*) Provide in the abstract an informative and balanced summary of what was done and what was found	2
**Introduction**			
Background/ rationale	2	Explain the scientific background and rationale for the investigation being reported	4
Objectives	3	State specific objectives, including any prespecified hypotheses	5
**Methods**			
Study design	4	Present key elements of study design early in the paper	6
Setting	5	Describe the setting, locations, and relevant dates, including periods of recruitment, exposure, follow-up, and data collection	6
Participants	6	(*a*) Give the eligibility criteria, and the sources and methods of selection of participants	6
Variables	7	Clearly define all outcomes, exposures, predictors, potential confounders, and effect modifiers. Give diagnostic criteria, if applicable	7-8
Data sources/ measurement	8*	For each variable of interest, give sources of data and details of methods of assessment (measurement). Describe comparability of assessment methods if there is more than one group	7-8
Bias	9	Describe any efforts to address potential sources of bias	6
Study size	10	Explain how the study size was arrived at	6
Quantitative variables	11	Explain how quantitative variables were handled in the analyses. If applicable, describe which groupings were chosen and why	8
Statistical methods	12	(*a*) Describe all statistical methods, including those used to control for confounding	8
		(*b*) Describe any methods used to examine subgroups and interactions	N/A
		(*c*) Explain how missing data were addressed	N/A
		(*d*) If applicable, describe analytical methods taking account of sampling strategy	N/A
		(*e*) Describe any sensitivity analyses	N/A
**Results**			
Participants	13*	(a) Report numbers of individuals at each stage of study—eg numbers potentially eligible, examined for eligibility, confirmed eligible, included in the study, completing follow-up, and analysed	8
		(b) Give reasons for non-participation at each stage	8
		(c) Consider use of a flow diagram	
Descriptive data	14*	(a) Give characteristics of study participants (eg demographic, clinical, social) and information on exposures and potential confounders	Table 1
		(b) Indicate number of participants with missing data for each variable of interest	N/A
Outcome data	15*	Report numbers of outcome events or summary measures	9-10
Main results	16	(*a*) Give unadjusted estimates and, if applicable, confounder-adjusted estimates and their precision (eg, 95% confidence interval). Make clear which confounders were adjusted for and why they were included	9-10
		(*b*) Report category boundaries when continuous variables were categorized	Tables 3-5
		(*c*) If relevant, consider translating estimates of relative risk into absolute risk for a meaningful time period	N/A
Other analyses	17	Report other analyses done—eg analyses of subgroups and interactions, and sensitivity analyses	N/A
**Discussion**			
Key results	18	Summarise key results with reference to study objectives	11
Limitations	19	Discuss limitations of the study, taking into account sources of potential bias or imprecision. Discuss both direction and magnitude of	12

For peer review only - http://bmjopen.bmj.com/site/about/guidelines.xhtml

		any potential bias	
Interpretation	20	Give a cautious overall interpretation of results considering objectives, limitations, multiplicity of analyses, results from similar studies, and other relevant evidence	13
Generalisability	21	Discuss the generalisability (external validity) of the study results	12-13
**Other information**			
Funding	22	Give the source of funding and the role of the funders for the present study and, if applicable, for the original study on which the present article is based	14

*Give information separately for exposed and unexposed groups.

Note: An Explanation and Elaboration article discusses each checklist item and gives methodological background and published examples of transparent reporting. The STROBE checklist is best used in conjunction with this article (freely available on the Web sites of PLoS Medicine at http://www.plosmedicine.org/, Annals of Internal Medicine at http://www.annals.org/, and Epidemiology at http://www.epidem.com/). Information on the STROBE Initiative is available at www.strobe-statement.org.

For peer review only
